# The effect of subject measurement error on joint kinematics in the conventional gait model: Insights from the open-source pyCGM tool using high performance computing methods

**DOI:** 10.1371/journal.pone.0189984

**Published:** 2018-01-02

**Authors:** Mathew Schwartz, Philippe C. Dixon

**Affiliations:** 1 Digital Human Research Center, Advanced Institutes of Convergence Technology, Seoul National University, Suwon, South Korea; 2 College of Architecture and Design, New Jersey Institute of Technology, Newark, NJ, United States of America; 3 Department of Environmental Health, Harvard T.H. Chan School of Public Health, Boston, MA, United States of America; 4 Liberty Mutual Research Institute for Safety, Hopkinton, MA, United States of America; Universitat Zurich, SWITZERLAND

## Abstract

The conventional gait model (CGM) is a widely used biomechanical model which has been validated over many years. The CGM relies on retro-reflective markers placed along anatomical landmarks, a static calibration pose, and subject measurements as inputs for joint angle calculations. While past literature has shown the possible errors caused by improper marker placement, studies on the effects of inaccurate subject measurements are lacking. Moreover, as many laboratories rely on the commercial version of the CGM, released as the Plug-in Gait (Vicon Motion Systems Ltd, Oxford, UK), integrating improvements into the CGM code is not easily accomplished. This paper introduces a Python implementation for the CGM, referred to as pyCGM, which is an open-source, easily modifiable, cross platform, and high performance computational implementation. The aims of pyCGM are to (1) reproduce joint kinematic outputs from the Vicon CGM and (2) be implemented in a parallel approach to allow integration on a high performance computer. The aims of this paper are to (1) demonstrate that pyCGM can systematically and efficiently examine the effect of subject measurements on joint angles and (2) be updated to include new calculation methods suggested in the literature. The results show that the calculated joint angles from pyCGM agree with Vicon CGM outputs, with a maximum lower body joint angle difference of less than 10^-5^ degrees. Through the hierarchical system, the ankle joint is the most vulnerable to subject measurement error. Leg length has the greatest effect on all joints as a percentage of measurement error. When compared to the errors previously found through inter-laboratory measurements, the impact of subject measurements is minimal, and researchers should rather focus on marker placement. Finally, we showed that code modifications can be performed to include improved hip, knee, and ankle joint centre estimations suggested in the existing literature. The pyCGM code is provided in open source format and available at https://github.com/cadop/pyCGM.

## 1 Introduction

### 1.1 Conventional gait model

Human locomotion has been a central theme of biomechanics research for centuries (c.f. [1] for a historical perspective). Fundamental to the evaluation of human motion is the precise quantification of three-dimensional joint kinematics (angles); however, as the technology to record motion grew over time, the translation between engineering and clinical approaches to measure this motion became a challenge [2]. Biomechanical models with clinical relevant measures have been developed to overcome this problem.

There are many biomechanical models available for kinematic analyses [3–8]. Of these, the CGM, also known as the Newington, Davis, Gage, Helen Hayes, or Kadaba model, has been used extensively in clinical and research settings for many years. More specifically, Vicon’s CGM implementation via the plug-in-gait modeler [9] is popular as it is distributed in their software packages such as Nexus [10]. As with many other models, the CGM relies on retro-reflective skin mounted markers capable of tracking body movements in order to compute joint kinematics [11]. This paper focuses on the Vicon CGM implementation. This model began to be defined by Kadaba, first with experiments in repeatability [8] and then as a method to calculate lower-limb joint angles [12], based on the orthopedic knee angle definitions of Grood and Suntay [13].

Although the CGM has been validated, it is not without problems. Directly related to the calculation methods of the CGM, four main issues appear. (1) As a direct kinematic and hierarchal method, a proximal origin’s frame definition influences more distal segments [12, 14]. For example, the definition of the tibia local coordinate system (distal) relies on the correct definition of the femur (proximal) local coordinate system. (2) Incorrect placement of markers along predefined anatomical landmarks is a known source of error in the CGM joint kinematic outputs [14–16]. (3) As with other marker-based models, the CGM is prone to errors from skin movement artifacs [17–19]. (4) The definition of the hip joint center location relies on regression equations from the early work of Davis et al. [20]. In practice, another issue has been the location of the knee and ankle joint centers having been generated by a proprietary formula known as the “Chord function” [9]. Improved approaches have been suggested in the literature (c.f. Harrington et al. [21] for refined regression equations for hip joint center identification and Stief et al. [22] for improved methods to identify the locations of the knee and ankle joints), but have not been natively implemented into the CGM model. An open-source CGM distribution could allow researchers to refine model outputs using these, or any other method described in the literature. Finally, the CGM maintains a low marker count (full kinematic analysis possible using 16 markers on the lower body and 19 markers on the upper body) through the input of subject anthropometric measurements and a static calibration trial, both of which allow previously defined quantities to act as virtual markers during the computation of joint kinematics. This approach introduces subject measurements as another potential source of error.

Previous work has shown that subject measurement error can propagate to joint kinematic quantities [23]. In the work of Benedetti et al. [23], the analysis of a single healthy subject at 7 different laboratories resulted in inter-laboratory differences of 30 mm in pelvic width (inter anterior-superior iliac spine distance), 25 mm in leg length, 5 mm in knee width, and 10 mm in ankle width measurements. The extent to which subject measurement error can affect joint angles has not been thoroughly explored. This last problem can be systematically investigated via the implementation of high performance computing methods in pyCGM.

### 1.2 Computational power and parallelization

Computational power has been continuously increasing for decades [24]. Through these advancements, numerous problems that were previously unsolvable due to the length of time needed for computations have been tackled using High Performance Computers (Computing) (HPC). Likewise, parallelization, in which multiple calculations can be executed simultaneously on different computer cores, especially when dealing with large numbers of independent variables, has been vital to understanding complex systems.

In the case of kinematic analysis, recording motions at high sampling rates over long periods of time can result in a significant amount of data to be processed, suggesting an important use-case for HPC and parallelization solutions. For example, a study on the effect of high heels on gait, in which 10 subjects walked at 5 cadences with 3 different heel heights, resulted a total of 150 trials to be analyzed [25]. For analyses of dynamic stability during gait, experiments often require considerably more data. In the work of Bruijn et al. [26], data were collected for 9 subjects during trials of 10 to 20 minutes. Furthermore, research has shown the usefulness of parallelization for auto-labeling of marker sets [27].

Currently, open source biomechanics tools such as OpenSim [28] and BTK [29] provide a platform for motion capture data visualization and biomechanics calculation. Additionally, the CGM has been implemented by [30]; however, the source code is not readily available and the literature does not discuss computational times and methods. Accessible open source and easy to modify code for joint kinematics is lacking, making it difficult for researchers to take advantage of the long history of motion capture and joint kinematic research for application in their own work. Recently Vicon Nexus has released an API to use their CGM, although this still requires a Vicon Nexus license. However, open source code that is only concerned with an input of marker locations disconnects it from any commercial software. This disconnect allows for identical calculations of joint kinematics from any hardware system (ex. Qualisys, Vicon, OptiTrack). For example, a user can leverage the Qualisys API to send marker data to pyCGM for kinematic calculations. Similarly, pyCGM could be used in conjunction with OpenSim to simulate and visualize movement. Without readily accessible code, each user must port biomechanical models to his or her own system, or rely on the real-time streaming ability of commercial products. For example, in robotics, research has been done on translating human motion to humanoid robots [31–33] and on using human motion capture data and analysis to work with humanoids through the development of HuMoD, an open database of a variety of gait motions and related measurements [34], largely in isolation from the developments in the gait community. The literature lacks exploration into the role of HPC and parallelization in terms of computational performance of joint kinematic analysis algorithms, such as with the CGM. At the same time, the introduction of HPC to the masses, such as Amazon Web Services, allows an easy platforms for development [35].

### 1.3 Python CGM

The basis of this paper is an open source Python script for the CGM, referred to as pyCGM [36]. First, a validation of the pyCGM joint kinematic outputs against the CGM model implemented by Vicon (Vicon Motion Systems Ltd., Oxford, UK) is presented. Second, the non-optimized direct kinematic approach of pyCGM allows for a frame-by-frame calculation for joint kinematics and, as such, provides an opportunity for easy HPC implementation to assist in the kinematic processing. Similarly, the portability and modularity provided by pyCGM allows researchers to quickly modify the parameters of the model and distribute workloads of either frame-by-frame or trial-to-trial calculations to an HPC. Thus, the reduction in joint kinematic computation times using pyCGM through HPC and parallelization approaches are explored. Third, the HPC setup is used to systematically explore the effect of subject measurement error on joint kinematics. Finally, examples of how pyCGM can be modified to include improvements to the CGM suggested in the literature are provided.

## 2 Methods

### 2.1 pyCGM process

**Fig 1:**
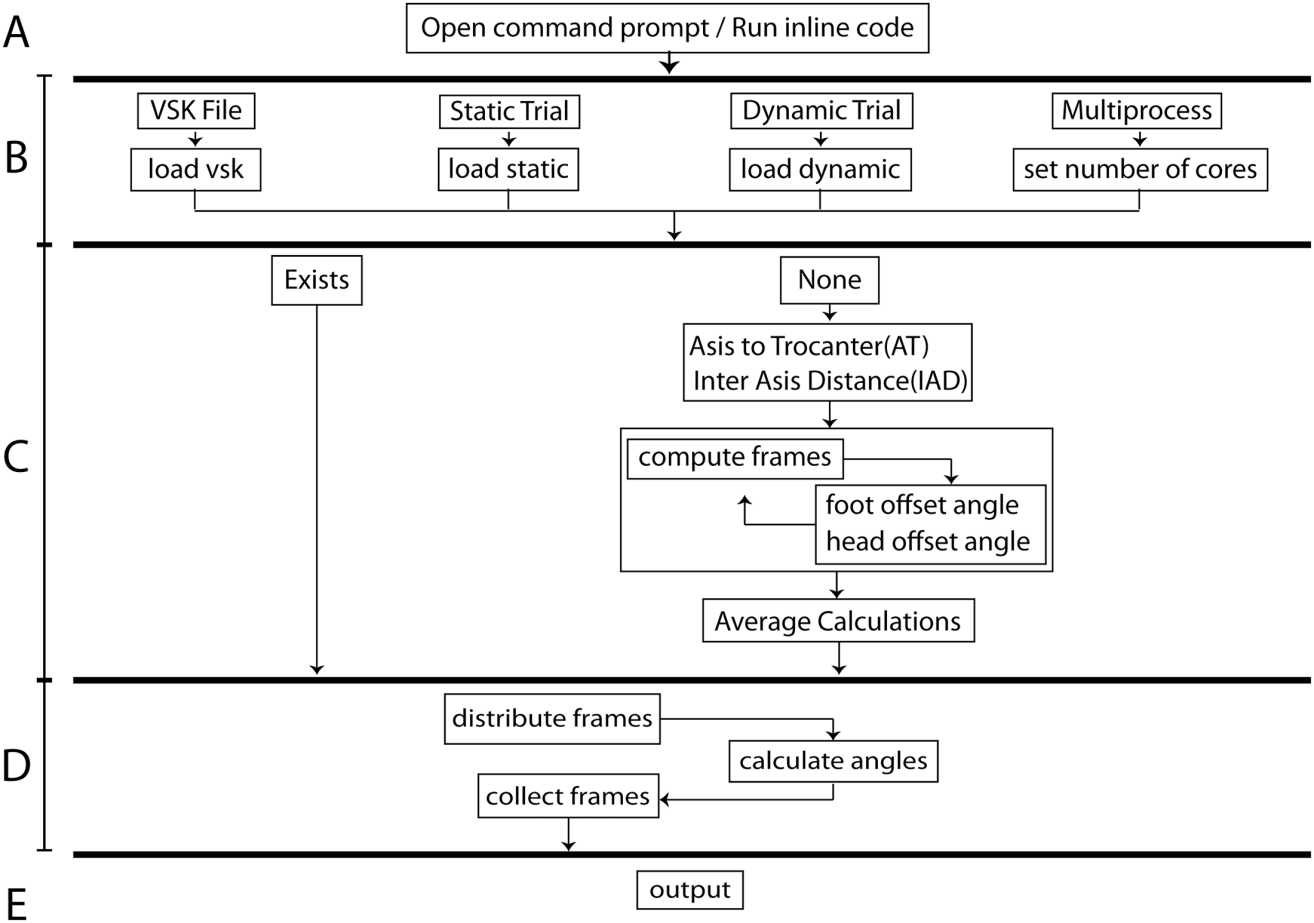
Process of the pyCGM system. There are five key parts to the computation. (A) A python terminal is passed arguments or the pyCGM is integrated into a larger python code. (B) Data is loaded for subject measurements, static and dynamic trials, and optional input for the number of cores to use. (C) If static offsets have not been provided, they are calculated using the static trial; otherwise, the calculation is passed. (D) Frames of the dynamic trial are divided and distributed to the number of specified cores. Subject measurements and static offsets are passed to each core as well. The results are gathered by the original process. (E) Data is saved in a user-specified format.

The pyCGM code can be run through either a command line or by directly calling the functions. Data can be passed to the joint angle calculations directly or by functions loading files which store data as a python dictionary. The process for calculating a dynamic trial is detailed in Fig 1.

Files are loaded or data is created in section B, with an argument passing the number of cores to use for calculations. Subject measurements can be loaded through a.VSK file (Vicon Skeleton), which may contain either subject measurements or subject measurements and corresponding static calibration information. Joint angles of a static trial can be obtained by using the same file for the dynamic trial. In section C, the static offsets are calculated when this information is missing. Static offsets rely on the computation of the CGM for each frame of the static trial and averaging the results at the end. Once the subject measurements and offsets are known, the frames of the dynamic trial are divided evenly among the number of cores defined and the data is distributed.

One of the key aspects to the CGM is estimating a joint center using subject measurements and virtual markers. While this method has been explained for the hip in [20], the authors are unaware of any paper that mathematically describes how the “Chord function” [9] computes knee and ankle joint centers. The calculation used here is with the Rodrigues rotation formula [37], as seen in S1 Equation. This formula allows for the minimum marker set by creating a virtual marker in the joint center that is used as the segment frame origin. The description is shown in S1 Fig, with the offset value being half of the knee width measurement, denoted by *kw*. As such, this joint center is directly dependent on the subject measurement. Implementation of this formula in pyCGM can be seen in the sample code shown in S2 Fig.

While pyCGM has followed the methods defined in the literature, one significant difference is implemented in the calculation of joint angles. While Kadaba [12] defined theta using arcsin, this only works up to a 90-degree rotation. As such, motions such as sitting in a chair or walking up stairs in which hips or knees will bend more than 90 degrees should be calculated using the arctan function, as seen in S3 Fig.

### 2.2 Parallelization

**Fig 2:**
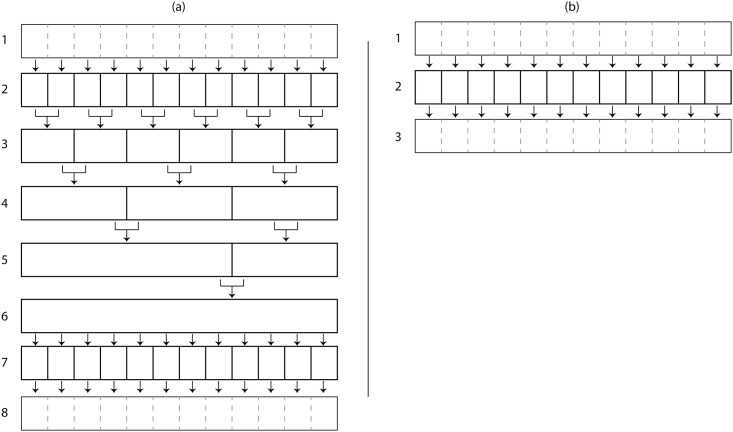
Abstraction of averaged calculation vs. direct calculation. (a) The first row shows data being passed to each theoretical core. Between rows 2 and 6, data is calculated in relation to itself, which reduces the maximum number of usable cores after each process. This method would be used to parallelize the static trial offsets, creating overhead between each data combination. (b) Calculations that are independent of the data can be calculated at once, making the maximum usable cores equal to the number of datum. This is the method used to parallelize the dynamic trial calculations.

The CGM has three main parts: a static trial, dynamic trial, and subject measurements. While a static calibration is required, the calculated values act as constants in the calculations. This independence is important for the parallelization efficiency and scalability of the system. The process requiring an average or other calculation over all frames can be seen in Fig 2(a), compared to Fig 2(b), in which each frame is independent.

While both Fig 2(a) and 2(b) begin with individual frames in row 1, Fig 2(a) row 2 must collect results from each process and pass them for further processing in row 3. Each time this occurs, an overhead for communication between processes is created. The static calibration of the CGM averages the offset angles of the ankle and neck, and averages the distance between ASIS markers. For this reason, the short static trials used in static calibration in this research are not parallelized and are instead calculated sequentially on a single core. However, the longer and frame-independent dynamic trials are the focus of the parallelization due to the higher increase in performance through optimal use of cores and low overhead, similar to Fig 2(b). In a similar way, an increase in frames requires a linear increase in computation, giving the method in Fig 2(b) a computational complexity O(n). In terms of computational time, an *n* increase in frames can be mitigated with an *n* increase in processors, with an overhead consistent with the communication algorithm.

#### 2.2.1 Desktop parallelization

**Fig 3:**
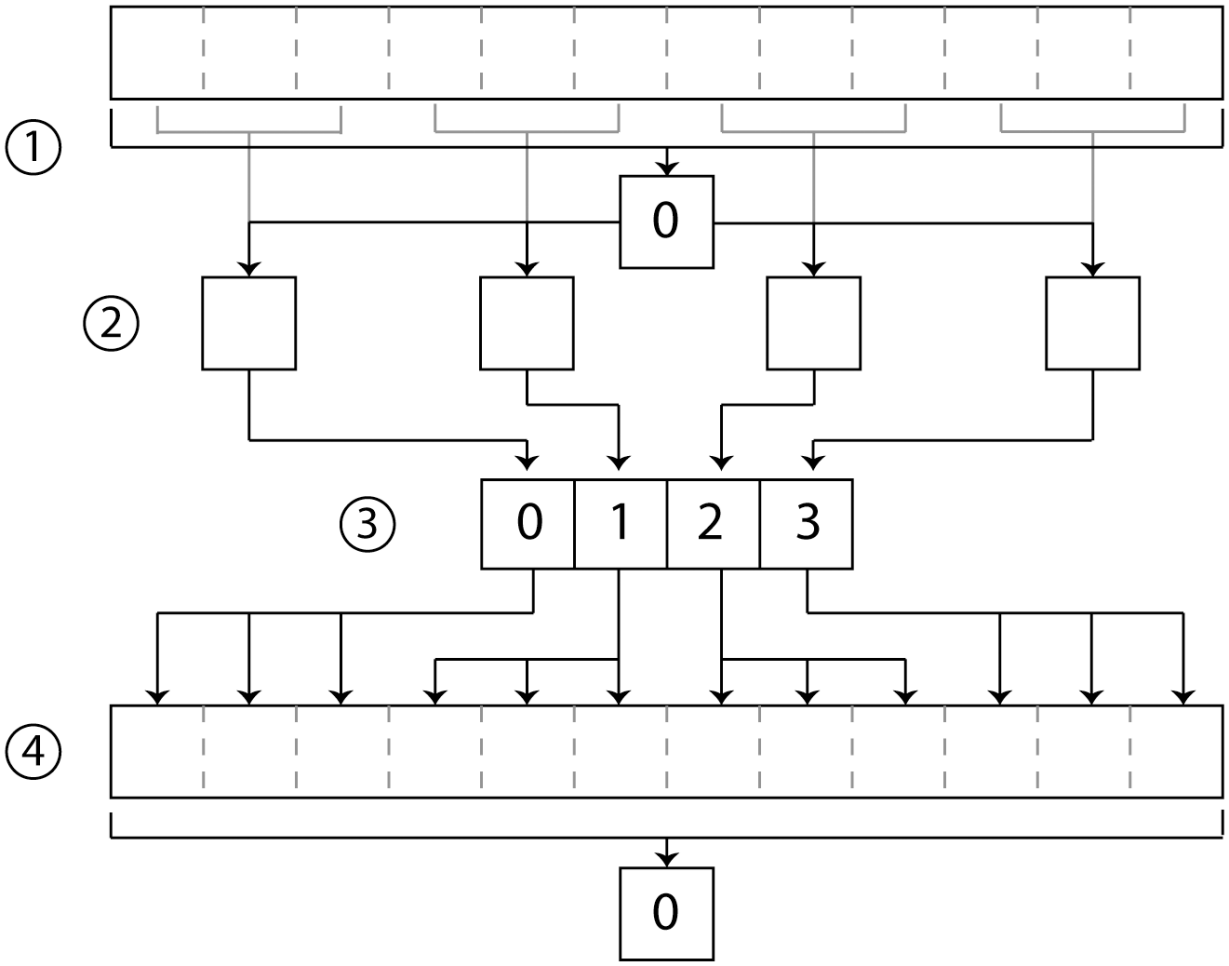
Diagram of parallelization for desktop. (1) Data is loaded by the root processor. (2) The root processor divides the data by the number of processes available and writes temporary files to memory. (3) The processes receive the file location containing their data and calculate the joint angles. (4) The root process gathers the data and each spawned process exits.

While access to HPC is increasing, the use of multi-core desktop computers is nearly ubiquitous, and as such, methods for parallelizing the CGM are applicable on these platforms as well. For simple cross-platform testing, the python multiprocessing module was used for parallel processing of dynamic trials (Fig 3). The original process on core 0 reads the required data such as dynamic trial, static offsets, and subject measurements (1). The data is stored as a python dictionary, which is split between keys and values for fast IO communication. Data are divided between frame length and number of cores to be used, then written as a temporary file to memory using mmap (2) while passing the file location to each process, which has a lower overhead than full use of serial communication directly between processes. The filename, memory size, and object size are stored, then later read and combined by each process (3) for use in the joint angle calculation. The multiprocessing.Process (an object of the multiprocessing library) method of python is used to instantiate a new instance of python for the calculation. After the result is put and aggregated by the original process, the open processes are exited(4).

#### 2.2.2 High performance computation

**Fig 4:**
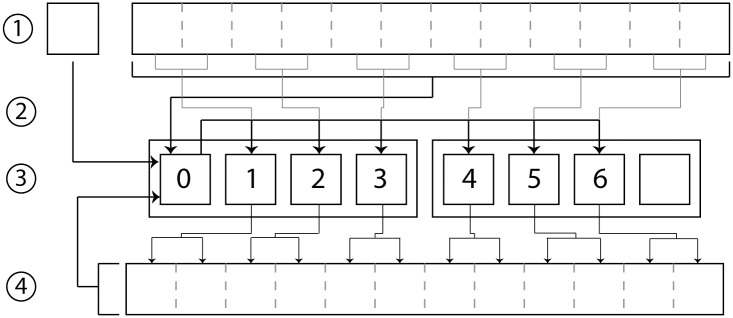
Abstraction diagram of a parallelized dynamic trial calculation on the HPC. In this example, processors 0-3 are located on node 1, and 4-6 are located on node 2. (1) The static trial, subject measurements, and the dynamic trial are stored on the IO system which are loaded by rank 0. (2) Rank 0 then distributes the frames evenly among the available ranks using the MPI.scatter method, and uses the MPI.bcast method to send the calculated static offsets and loaded subject measurements. (3) Rank 0 collects the joint angle results with the MPI.gather method.

Similar to the method for desktop parallelization, a single dynamic trial can be distributed among multiple nodes on an HPC. In this method, an initial processor (rank 0) is responsible for loading and distributing data (Fig 4). Rank 0 loads subject measurements, the static trial, and the dynamic trial. The static offsets are calculated and the dynamic trial is divided into dictionary labels and values, which are distributed among the number of ranks available along with the static information.

**Fig 5:**
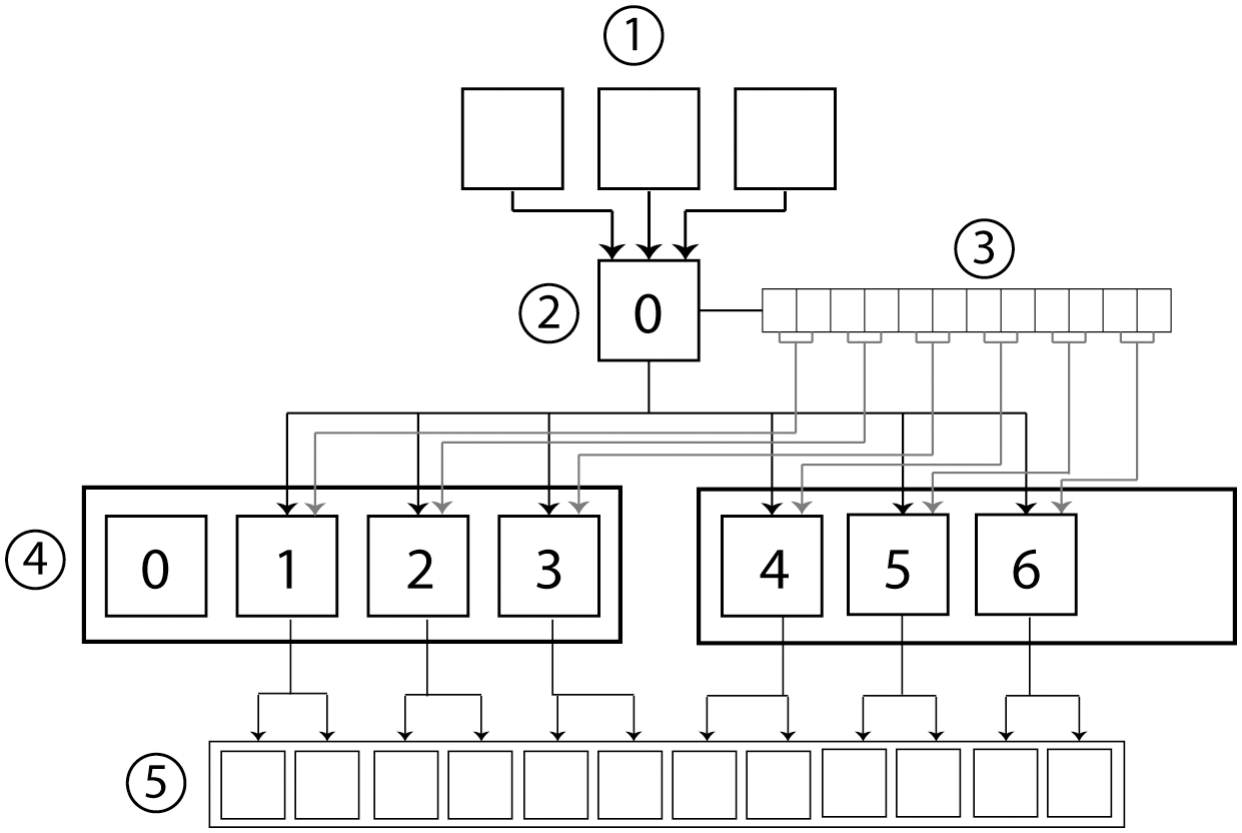
Abstraction diagram of distributed computation of multiple files on the HPC. (1) The dynamic trial, static trial, and subject measurements are stored on the IO Luster system. (2) The rank 0 core loads the data from the luster system. (3) Rank 0 calculates all variations of subject measurements and distributes them over the available ranks using the MPI.scatter method. (4) Rank 0 uses MPI.bcast method to distribute the dynamic trial, static trial, and subject measurements to all ranks. (5) Each rank saves the resulting data directly to the luster system.

Calculation of multiple trials or, in the case of this research, multiple iterations of a single dynamic trial can be done by either passing data using MPI or by relying on the IO luster system. In Fig 5, the method for distributing data using MPI is detailed. Rank 0 loads subject measurements, the static trial, and the dynamic trial. In addition, an array of subject measurement offsets is equally divided among the number of ranks used in the calculation. Rank 0 uses MPI scatter to distribute the offsets among ranks, and uses bcast to distribute the static trial, subject measurements, and dynamic trial to all ranks involved in the calculations. Each rank then uses the given array of subject measurement offsets to recalculate the static trial and calculate the new joint angles from the dynamic trial. Each rank saves the calculated joint angles and iterates through the array of subject measurements. In this system, rank 0 does not handle the gathering or saving of the data from each rank, and instead tracks computation time and when all ranks have finished calculations.

### 2.3 Data

For analyzing the computational performance of a parallel system on large motion capture data, a file containing 59,993 frames was recorded at 100 Hz in Vicon Nexus (v1.8, Vicon Motion Systems Ltd, Oxford, UK) using the 35 marker set. This trial is used purely as a method for checking parallelization efficiency and space complexity, and not used for the subject measurement analysis.

To analyze the effect of subject measurement on joint kinematics, the sample data distributed on Vicon Nexus systems was used for its wide availability and unbiased marker positioning. This dataset includes, among others, a static trial with 275 frames, a range of motion (ROM) trial with 2,076 frames, and complete subject measurements stored in a.VSK file, all three of which are used in this research. The trials contain two additional markers on each arm, UPA and FRM; however, these markers are not used in the calculations. The ROM file was selected for analysis, as it allows for a thorough understanding of the influence of subject measurements throughout different motions that may be calculated by the CGM. For example, hip flexion that occurs during a sitting motion would not appear during a typical gait cycle. While most of the data was complete, the squat and bending motion of the ROM had occluded markers which were gap-filled within the Vicon Nexus software. The ROM file data was categorized into the individual movements for analysis. The data displayed in the Results includes a squat motion, front leg raise, and a side leg raise.

Saving the results is done with the numpy compressed format NPZ. The advantage of the NPZ format over C3D format is the fast data IO and extremely simple programming methods. Keywords are used to determine which of the joint angles are to be saved in the file to reduce unnecessary time and space in the saving process. In the case of this research, the joint angles for only the lower body are saved in the NPZ format(S1 File). Table 1 shows the space required to save various numbers of frames.

**Table 1:** Space requirement for saving lower body joint angles and axis to a .npz file.

Frames	.npz Angles and Axis	.npz Angles	.c3d Without UPA/FRM	.c3d With UPA/FRM
**100**	81,884	13,672	61,632	82,080
**1000**	827,094	137,901	565,632	751,680
**10000**	8,397,657	1,410,613	5,605,632	
**50000**	42,016,679	7,070,983	Nexus Failed Saving	

Values are in bytes. The Angles and Axis column represents the file size when both angles and the frames used to calculate the angles are saved. The Angles column is when only the angles are saved. In both cases, joint angle data is for right and left, hip, knee, ankle, and foot progression angles. The sample data from Vicon Nexus includes the UPA and FRM markers, however, the trial used for longer calculations does not.

### 2.4 Subject measurements

There are 6 subject measurements related to distance that affect the joint angle calculations of the lower body: ASIS to trochanter distance (AT), inter ASIS distance (IAD), leg length (LL), knee width (KW), ankle width (AW), and sole delta. While LL, KW, and AW are required inputs for the CGM, the AT and IAD can either be input manually or calculated automatically from marker positions, and sole delta can be set to 0. As the former three are required in all situations, the current analysis is focused on these parameters. To demonstrate the HPC performance of numerous dynamic trials, various permutations of these 5 measurements are calculated. Permutations with repetitions are calculated by the *n* number of possible subject measurement values to the power of *r* number of measurements, denoted by *n*^*r*^. Sampling of the values was done in three steps.

- Intervals of 0.5 from -5 to 5 are generated for LL, KW, and AW, resulting in 21^3^ combinations.
- A large range from +/- 0 to 80 for LL, KW, and AW with increments doubling for 9^3^ combinations.
- An additional 9^3^ combinations involving AT and IAD were used for the computational performance experiment, but not used for data analysis.
- Excluding duplicates from each range, such as all measurements being 0.

The total number of these combinations results in 10,685 unique dynamic trials being calculated. Both the analysis and computational times for calculating these files are detailed in the Results section.

### 2.5 Hardware and software

Experiments were done on two platforms using Python 2.7. First, the python multiprocessing module was implemented on a laptop running Windows 7 64-bit with a quad core Intel i7-4700MQ processor at 2.4 GHZ, and 16 GB of RAM. This laptop was also used for the timing experiments to compare against Vicon Nexus 1.8. For the HPC experiment a Cray XC30 supercomputer was used (Referred to as Darter). Each node consists of two 2.6GHz Intel 8-core XEON E5-2600 CPUs and 32GB of RAM, with hyper-threading disabled.

## 3 Results

### 3.1 Kinematic validation

The dynamic ROM trial joint kinematic outputs from pyCGM and Vicon’s CGM implentation were compared (Table 2). The results show that both the upper and lower body joint angle estimations from pyCGM agree with the Vicon CGM outputs within 10^-5^ degrees.

**Table 2:** Joint angle differences between Vicon CGM and pyCGM for the ROM file.

**Lower**	**X**	**Y**	**Z**	**Maximum**
R Pelvis	2.66359E-06	3.31919E-06	4.27735E-06	**0.000004**
L Hip	6.41988E-06	-1.37459E-05	2.6727E-05	**0.000027**
R Hip	5.38463E-06	-1.08932E-05	5.17191E-05	**0.000052**
L Knee	1.1362E-05	3.41015E-05	1.14514E-05	**0.000034**
R Knee	1.07576E-05	4.18676E-05	1.25827E-05	**0.000042**
L Ankle	-1.55859E-05	8.80732E-06	1.40949E-05	**0.000014**
R Ankle	-2.91199E-06	2.5783E-05	1.54074E-05	**0.000026**
L Foot Progress	5.03701E-05	4.05533E-05	8.8887E-06	**0.000050**
R Foot Progress	2.77097E-05	1.27706E-05	9.09723E-06	**0.000028**
**Maximum**	**0.000050**	**0.000042**	**0.000052**	
**Upper**	**X**	**Y**	**Z**	
L Shoulder	4.26187E-06	5.26738E-06	1.16389E-05	**0.000012**
R Shoulder	4.27861E-06	5.75079E-06	6.06542E-06	**0.000006**
L Elbow	7.35977E-06	6.552E-12	6.551E-12	**0.000007**
R Elbow	8.83274E-06	7.974E-12	7.603E-12	**0.000009**
L Wrist	3.99468E-06	3.60593E-06	9.05932E-06	**0.000009**
R Wrist	2.37015E-06	3.46521E-06	1.08998E-05	**0.000011**
R Spine	2.88501E-06	3.3882E-06	3.33372E-06	**0.000003**
R Thorax	4.75358E-06	1.69255E-06	4.253E-06	**0.000005**
R Neck	8.83274E-06	7.974E-12	7.603E-12	**0.000009**
R Head	3.3013E-05	1.40494E-05	2.51925E-05	**0.000033**
**Maximum**	**0.000033**	**0.000014**	**0.000025**	

Values are calculated over the entire ROM file. Single joints such as the pelvis, spine, thorax, neck, and head output from pyCGM are compared against the “right side” corresponding angle as the Vicon CGM uses the same value for both sides.

### 3.2 Computational performance

**Fig 6:**
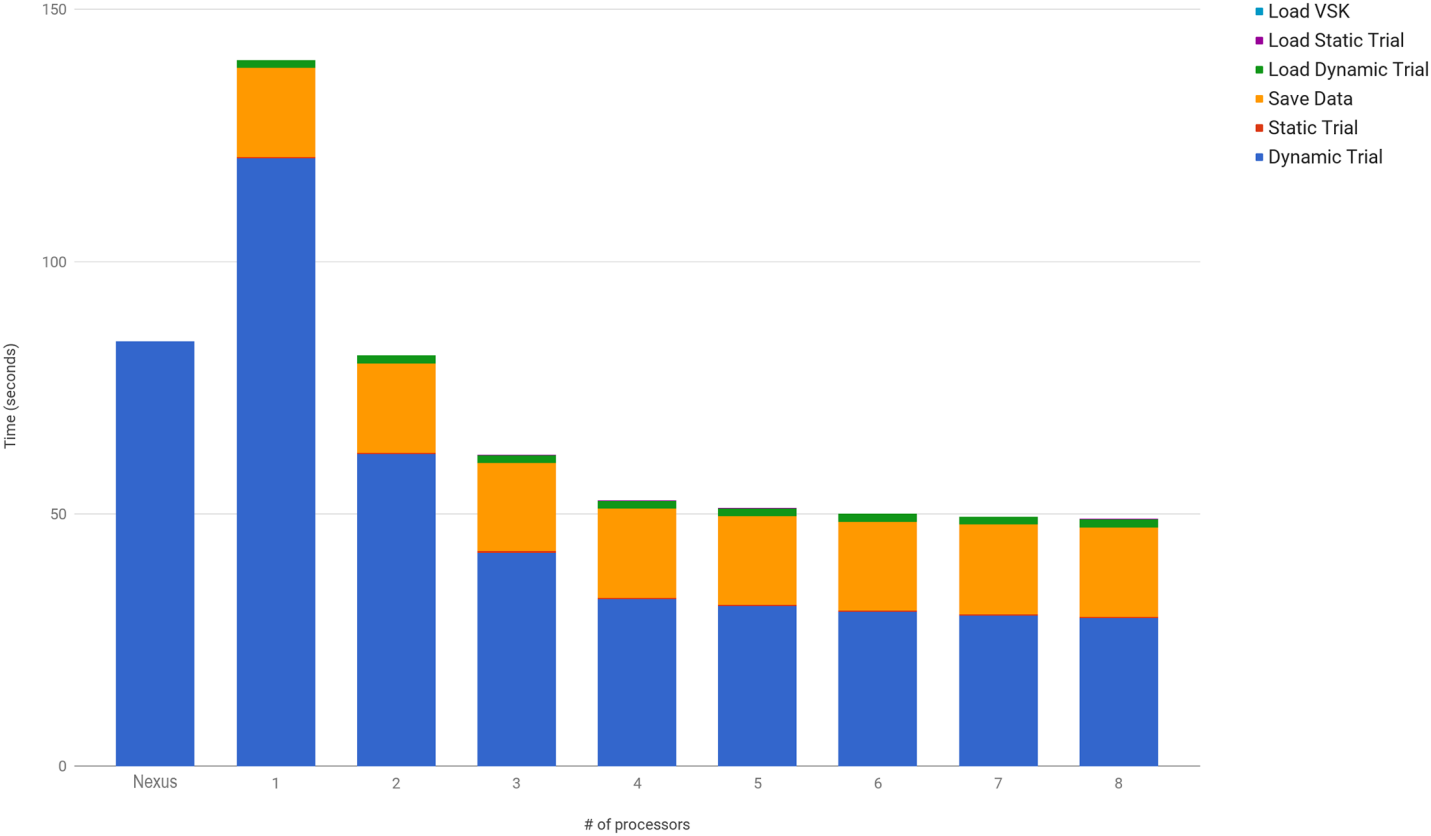
Computation time comparisons between Vicon’s CGM implementation (Nexus) and parallel programming of pyCGM (Python) on a consumer laptop for a trial containing 59,993 frames. Note: The laptop has 4 physical cores hyper-threading, but displays 8 processors to the multiprocessing module.

The first experiment comparing the computational performance of the pyCGM against Vicon’s CGM implementation in Nexus v1.8 is on a consumer-based four-core laptop. As Nexus calculates both kinetics and kinematics, it is not possible to compare exact computational speed differences against pyCGM. However, the results give a frame of reference for practical computation times on any multi-core consumer computer when kinetic calculations are not required. Fig 6 shows that with a four-core computer, the parallelized version of the CGM implemented in python can provide significant improvements to the calculation times specific for kinematics. As a stand-alone compiled program, comparison against Nexus’s computation time demonstrates the speed benefit of parallelization.

**Fig 7:**
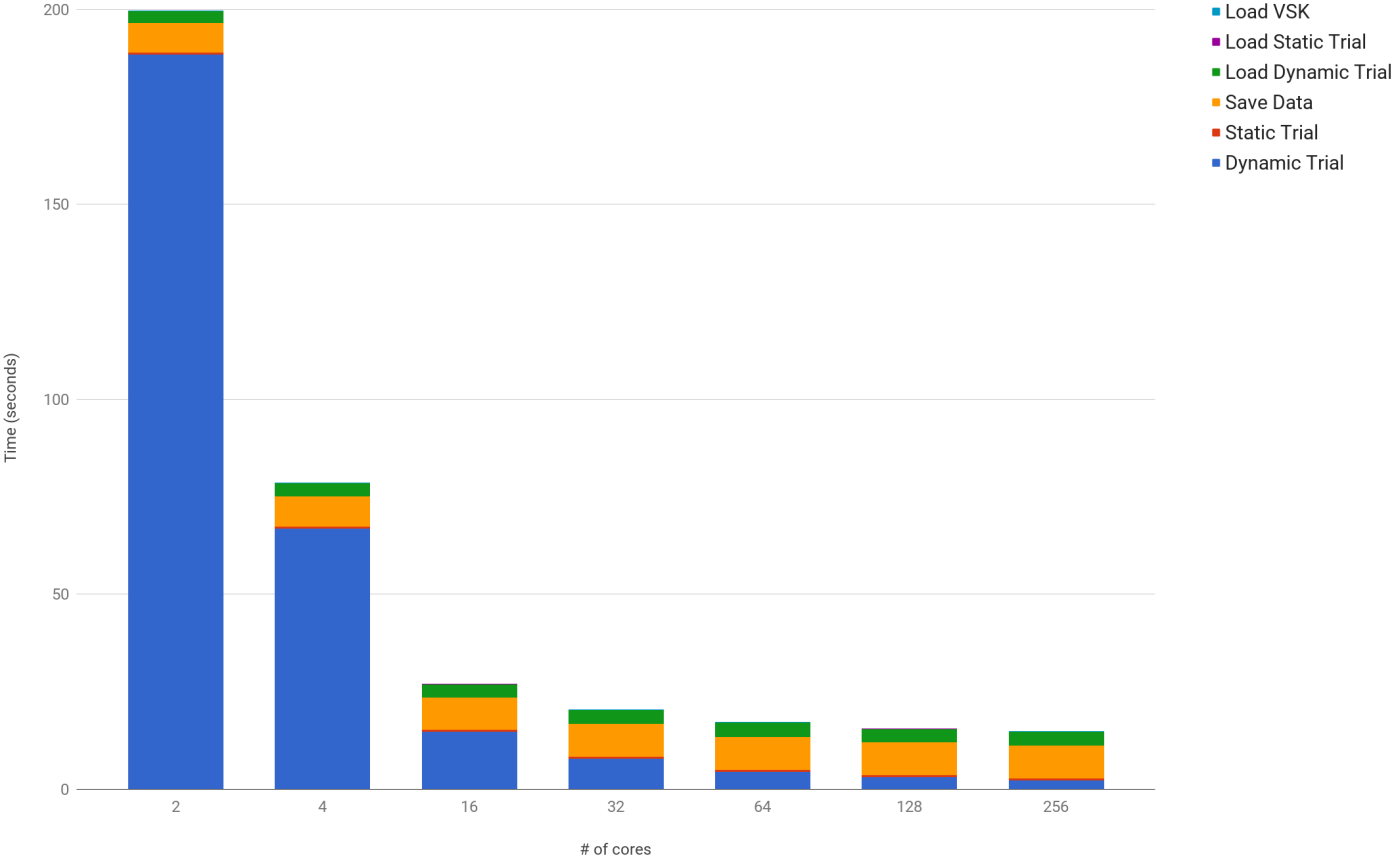
Computation time of a dynamic trial across multiple cores and nodes. While the dynamic trial scales close to ideal across multiple cores and nodes, the single core calculations and IO operations remain nearly constant, with small variations over each experiment. S1 Table provides a more detailed view of these results.

Scalability on the HPC of the multiple core approach to the CGM can be seen in Fig 7. While the dynamic trial is parallelized, the file IO and static calibration are not, and as such, these remain nearly constant, as shown in Fig 4. Furthermore, the efficiency in scaling the CGM can be seen in Table 3, as the actual increase in performance closely tracks the ideal. By utilizing 16 nodes, this implementation can calculate over 27,000 frames per second.

**Table 3:** Computational performance of kinematic calculations for a dynamic trial on the HPC.

Cores	Ideal	Actual	Frames/Second
2	188.4	188.4	318.5
4	62.8	66.8	897.9
16	12.6	14.8	4,063.2
32	6.1	7.9	7,608.1
64	3.0	4.5	13,435.0
128	1.5	3.0	19,734.4
256	0.7	2.2	27,011.8

Each node on the HPC consists of 16 cpu cores. One node was used for the calculations until 16 cores, after which the number of nodes increase. Although 2 cores are used to begin with, one core is reserved for managing data and recording times. This explains the jump in performance from 2 to 4 cores. As can be seen, the actual performance closely tracks the ideal performance of a parallelized model until 64 cores, after which the ideal metric outperforms the actual by approximately 2 to 1. By 256 cores, over 27,000 frames can be calculated per second.

**Fig 8:**
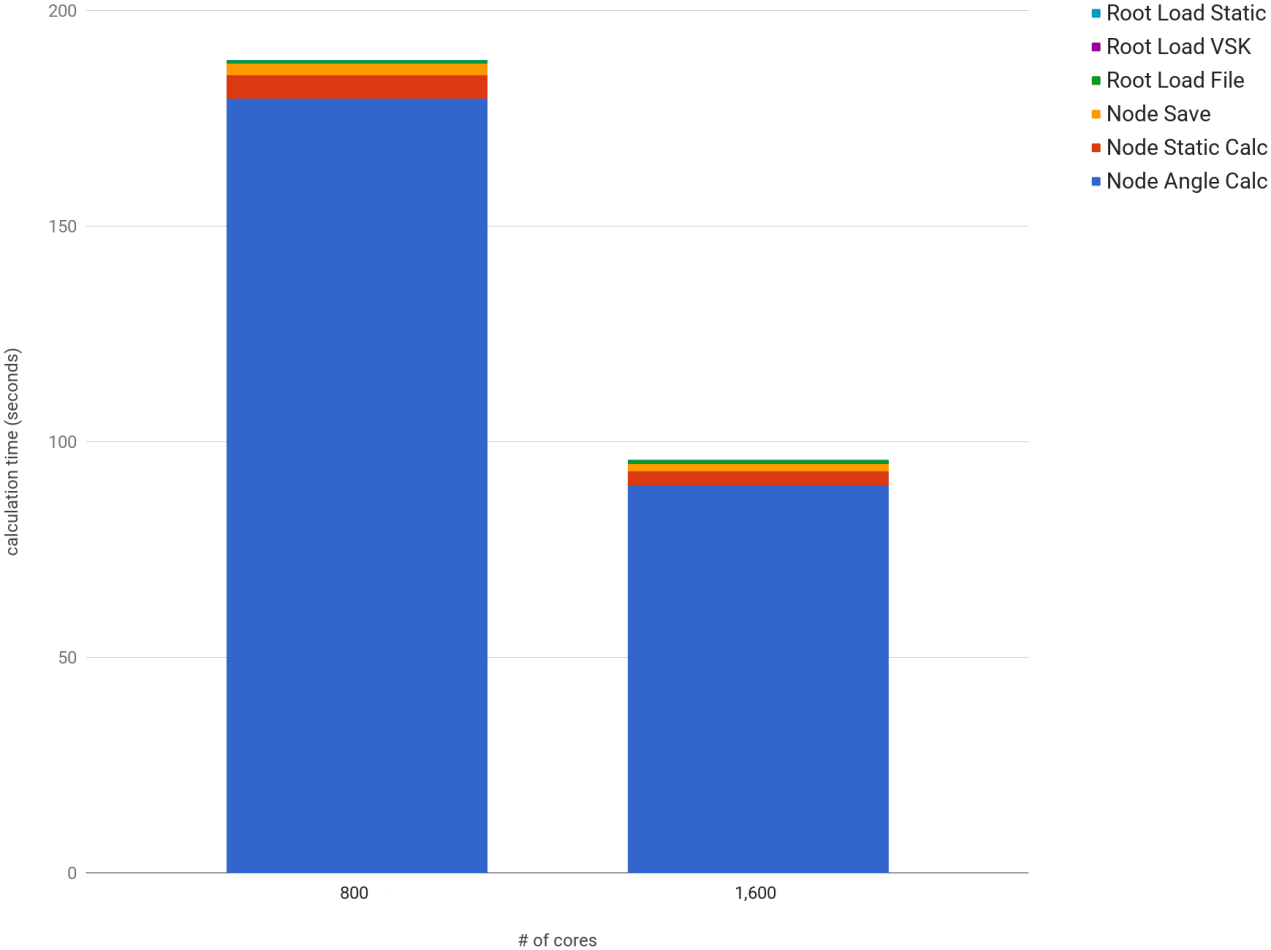
Computation time of multiple variations of the ROM trial. The sections prefixed with ‘Root’ refer to operations that occur once at the beginning of the experiment on the initial core. The ‘Node’ prefix refers to calculations that occur on each core, with the average values shown in the figure. The numeric results with minimum and maximum times are shown in S2 Table.

To calculate multiple variations of a dynamic trial, an experiment with both 800 and 1,600 cores was conducted using the method explained in Fig 5. Similar to the previous experiment, the increase in computing nodes scaled the computation time to a near ideal rate with the average core calculation time for 10,685 variations of the dynamic ROM trial (2,076 frames) with 800 cores at 179.6739 seconds, and 1,600 cores at 89.7404 seconds. The latter time demonstrates an overall computation speed of 247,180 frames per second. Fig 8 shows the calculation times on the initial core and the averaged times for calculations occurring across all cores.

### 3.3 Influence of subject measurements

Three key aspects of the relationship between subject measurement error and joint angle error were analyzed. First, the maximum joint angle error of a +/- 2.5% and +/- 5% error in leg length (LL), +/- 5% and +/- 10% in knee width (KW), and +/- 10% and +/- 20% in ankle width (AW) measurements during various motions from the ROM trial was found. The subject measurement percentage error corresponds to the errors found from inter-laboratory testing in [23]. Second, the joint angles of the original data, along with +/- 5% error in LL and KW are shown for a squat motion in the knee joint. Finally, the subject measurement variations are ranked by the largest error created for each combination, providing insights to the importance of each subject measurement and the predictability of this error.

As the CGM is based on a hierarchy of relative joints, the subject measurements influence the joint angles in this hierarchy. As such, the modification of LL affects all joints, while the modification of AW will only have a direct effect on the ankle frame, with an indirect effect of the knee kinematics. For both squat and ankle rotation motions extracted from the ROM file, Table 4 shows the wide range of joint angle error caused by changing each subject measurement independently. The data suggest that the rotation (z axis) in each joint is the most prone to error in a majority of cases. However, Knee abduction (y axis) is also greatly affected by subject measurement errors. Ankle non-sagittal angles (y and z axes) show large error, but are not often considered in clinical analysis using the CGM. Importantly, during a squat motion the flexion (x) axis was not the largest source of error. Additionally, the range of error between the minimum and maximum throughout the motion suggests that the error is not a fixed offset.

**Table 4:** Joint angle error from variations in subject measurements.

			LL		KW		AW	
			±2.5%	±5%	±5%	±10%	±10%	±20%
**Squat** **(3605∼3875)**	**Hip**	**Flexion(X)**	0.38	0.77	0.26	0.53		
		**Abduction(Y)**	0.23	0.46	0.36	0.73		
		**Rotation(Z)**	1.59	**3.16**	0.26	0.53		
	**Knee**	**Flexion(X)**	0.52	1.04	0.20	0.40	0.23	0.46
		**Abduction(Y)**	1.45	**2.91**	0.69	1.38	0.50	1.00
		**Rotation(Z)**	0.68	1.37	0.95	1.95	0.04	0.09
	**Ankle**	**Flexion(X)**	0.22	0.44	0.18	0.37	0.30	0.59
		**Abduction(Y)**	0.25	0.50	0.23	0.47	0.35	0.70
		**Rotation(Z)**	0.66	1.33	0.75	1.52	0.10	0.22
**Front Kick** **(2925∼2975)**	**Hip**	**Flexion(X)**	0.36	0.72	0.12	0.24		
		**Abduction(Y)**	0.24	0.48	0.37	0.74		
		**Rotation(Z)**	1.37	**2.75**	0.08	0.17		
	**Knee**	**Flexion(X)**	0.24	0.49	0.12	0.25	0.19	0.38
		**Abduction(Y)**	1.09	**2.18**	0.69	1.38	0.47	0.93
		**Rotation(Z)**	0.64	1.29	0.68	1.40	0.03	0.05
	**Ankle**	**Flexion(X)**	0.23	0.46	0.11	0.22	0.33	0.66
		**Abduction(Y)**	0.30	0.60	0.28	0.57	0.34	0.70
		**Rotation(Z)**	0.71	1.44	0.54	1.12	0.21	0.42
**Leg Swing** **(2200-2500)**	**Hip**	**Flexion(X)**	0.34	0.69	0.20	0.40		
		**Abduction(Y)**	0.28	0.56	0.36	0.72		
		**Rotation(Z)**	1.74	**3.47**	0.09	0.17		
	**Knee**	**Flexion(X)**	0.33	0.67	0.05	0.10	0.22	0.43
		**Abduction(Y)**	0.95	1.90	0.68	1.37	0.44	0.89
		**Rotation(Z)**	0.80	1.60	0.61	1.24	0.02	0.03
	**Ankle**	**Flexion(X)**	0.40	0.81	0.40	0.83	0.50	1.01
		**Abduction(Y)**	0.53	1.09	0.62	1.28	0.37	0.76
		**Rotation(Z)**	0.70	1.41	0.69	1.40	0.25	0.52

The original values for Leg Length (LL), Knee Width (KW), and Ankle Width (AW) are 940 mm, 105 mm, and 70 mm, respectively. As such a 5% change in LL is 47 mm, while the corresponding change for KW is 5.25 mm. The maximum values are combined from both the left and right joint angles and the positive and negative directions of the offset. The Cells left blank are due to adjustments in ankle width not affecting the Hip axis. Cells with Bold text are errors over 2 degrees.

**Fig 9:**
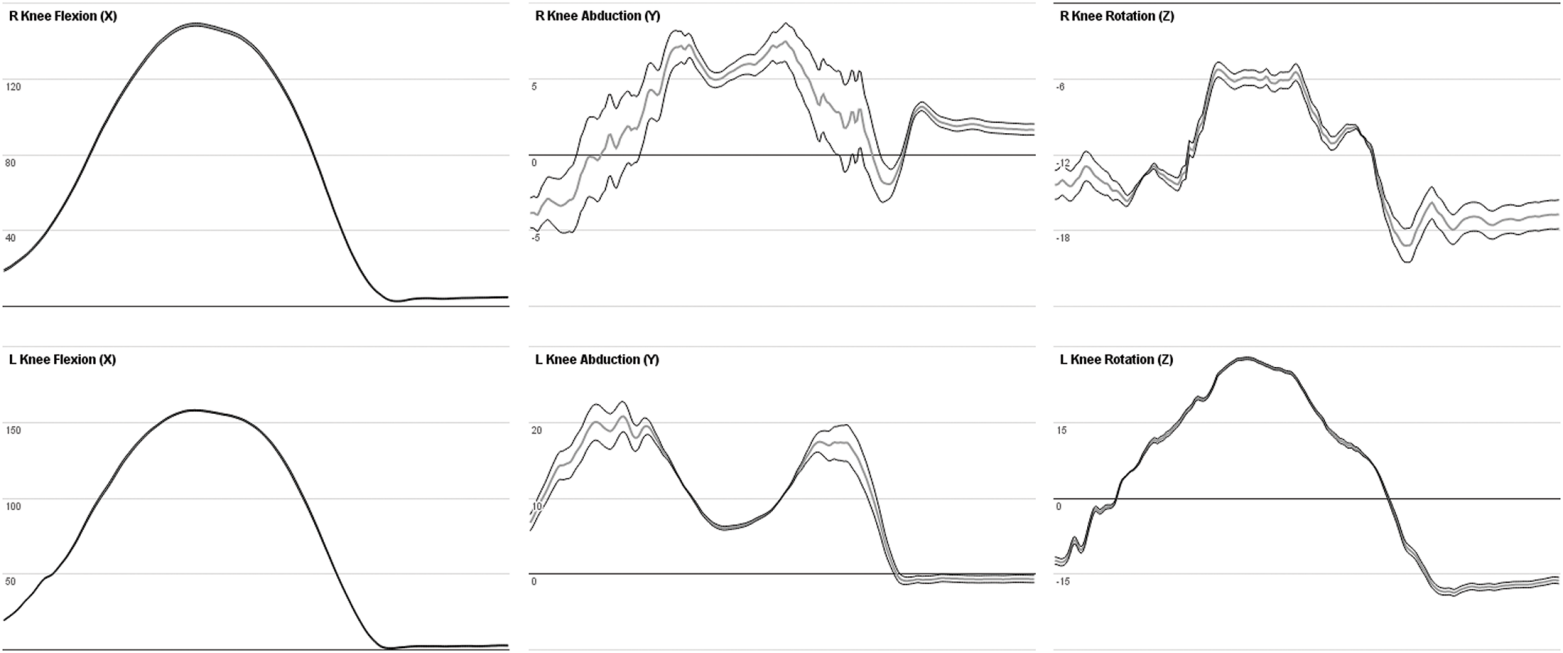
Joint angles of the knee during squat while changing the leg length. Graph of the right and left knee joint angles during a squat motion over 270 frames (3,605–3,875) of the ROM trial. Leg length was changed by +/-5%. The original angle is represented by the grey line and the leg length modifications are shown by the black line.

**Fig 10:**
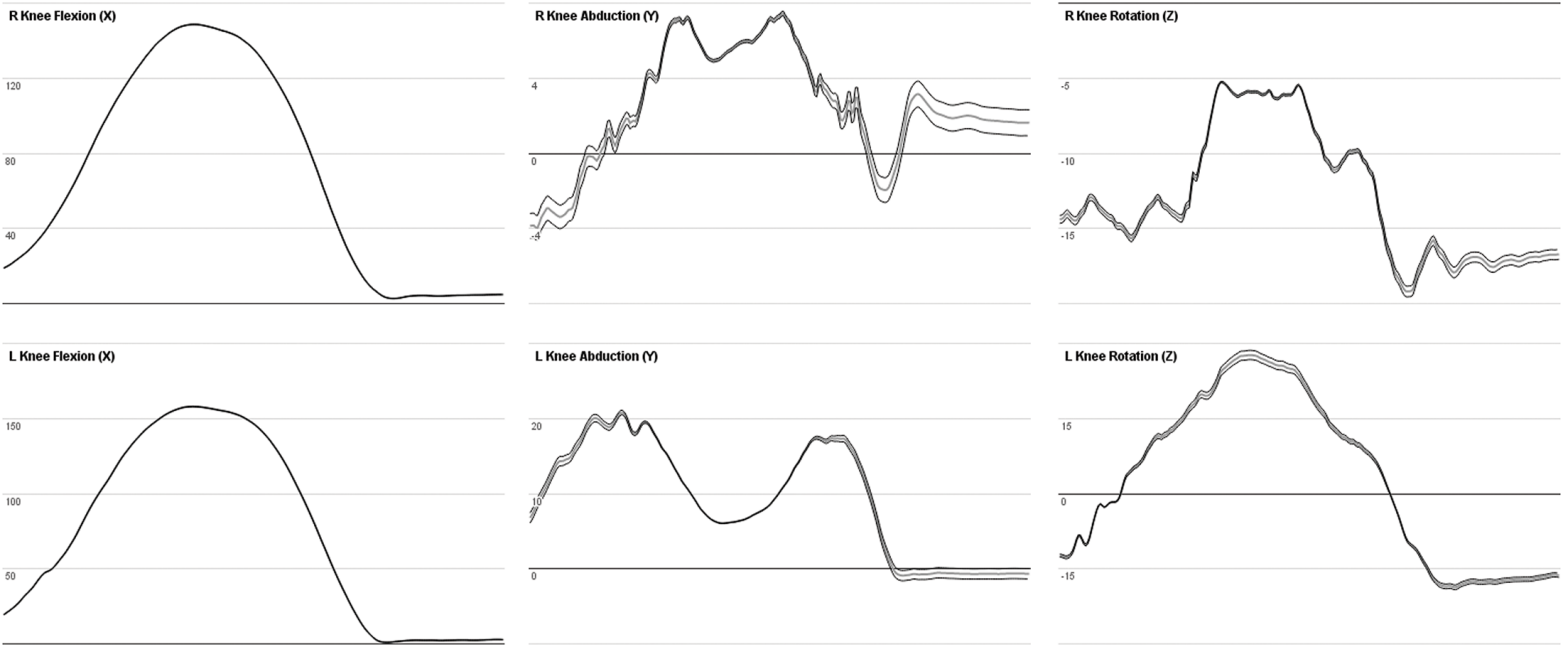
Joint angles of the knee during squat while changing the knee width. Graph of the right and left knee joint angles during a squat motion over 270 frames (3,605–3,875) of the ROM trial. Knee width was changed by +/-5%, where the original angle is represented by the grey line, and the knee width modifications in the black line.

The irregular effect of subject measurements on joint angles is due in part to both the calculation method (S1 Fig) and the hierarchal configuration. During motion such as a squat, the knee flexion axis is relatively unchanged, as this primary axis is defined by the lateral knee marker. Additionally, a change in the leg length measurement changes the hip joint center, which in turn changes the orientation of the knee rotation axis (z). Hence both the leg length (Fig 9) and knee width (Fig 10) measurements affect the knee joint angle in a different manner over the course of a motion.

Finally, by analyzing the 9,965 combinations of LL, KW, and AW, the most affected joint and axis can be found. Through the hierarchal system, the largest angle error of all combinations occurrs most often in the ankle joint (Table 5). The frame in which each joint incurs the largest error across all measurements is largely consistent within the individual joint, but varies across joints.

**Table 5:** Total number of subject measurement errors.

Total Joint	Joint Count	Number of Frames	Max Frame #	Max Frame Count
**Total**	9965	66	1919	2599
**Hip**	3736	20	1919	5464
**Knee**	1474	90	3773	5130
**Ankle**	4755	55	2672	2732

Across all frames and measurements, the Joint Count lists the number of times a particular joint contained the highest angle error, the Number of Frames counts the total number of unique frames which contained the highest angle error, the Max Frame # specifies which frame number contained the most frequent angle error, and the Max Frame Count is the number of times the corresponding frame appeared.

The large difference in the most common joint axis angles between right and left sides shows that the initial frame orientation and definition greatly influences the resulting errors from subject measurements(Table 6).

**Table 6:** Most frequent axis containing the largest angle error across all subject measurement variations.

Axis		Hip	Knee	Ankle
**Right**	**X**	27	1	7025
	**Y**	0	8333	1747
	**Z**	9200	28	1133
**Left**	**X**	0	0	60
	**Y**	675	265	0
	**Z**	63	1338	0

The total number of times that each axis was recorded containing the largest joint angle error across all subject measurement variations. A breakdown of these values can be seen in S2 File.

### 3.4 CGM modifications

The code style of pyCGM was developed to be straight forward to understand and modify. Beyond the utility of being cross platform without modification there are no pointers or objects used in the calculation. Subject measurements are stored in a dictionary and are easily referred to throughout the code.

**Fig 11:**
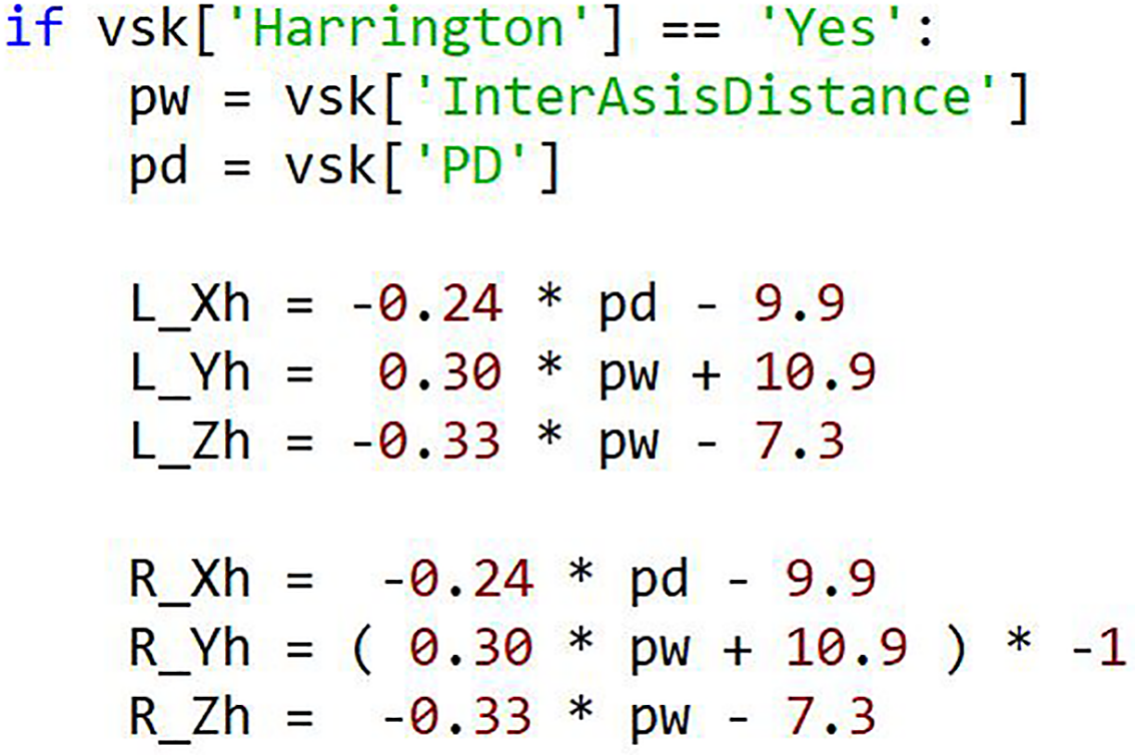
Implementation of Harrington method. The use of a keyword setting Harrington to True activates the if statement to use the Harrington Hip Regression Method.

Improvement of the original CGM hip joint center location estimations [20] were implemented based on the work of Harrington et al. [21]. Fig 11 shows the minimal amount of code.

**Fig 12:**
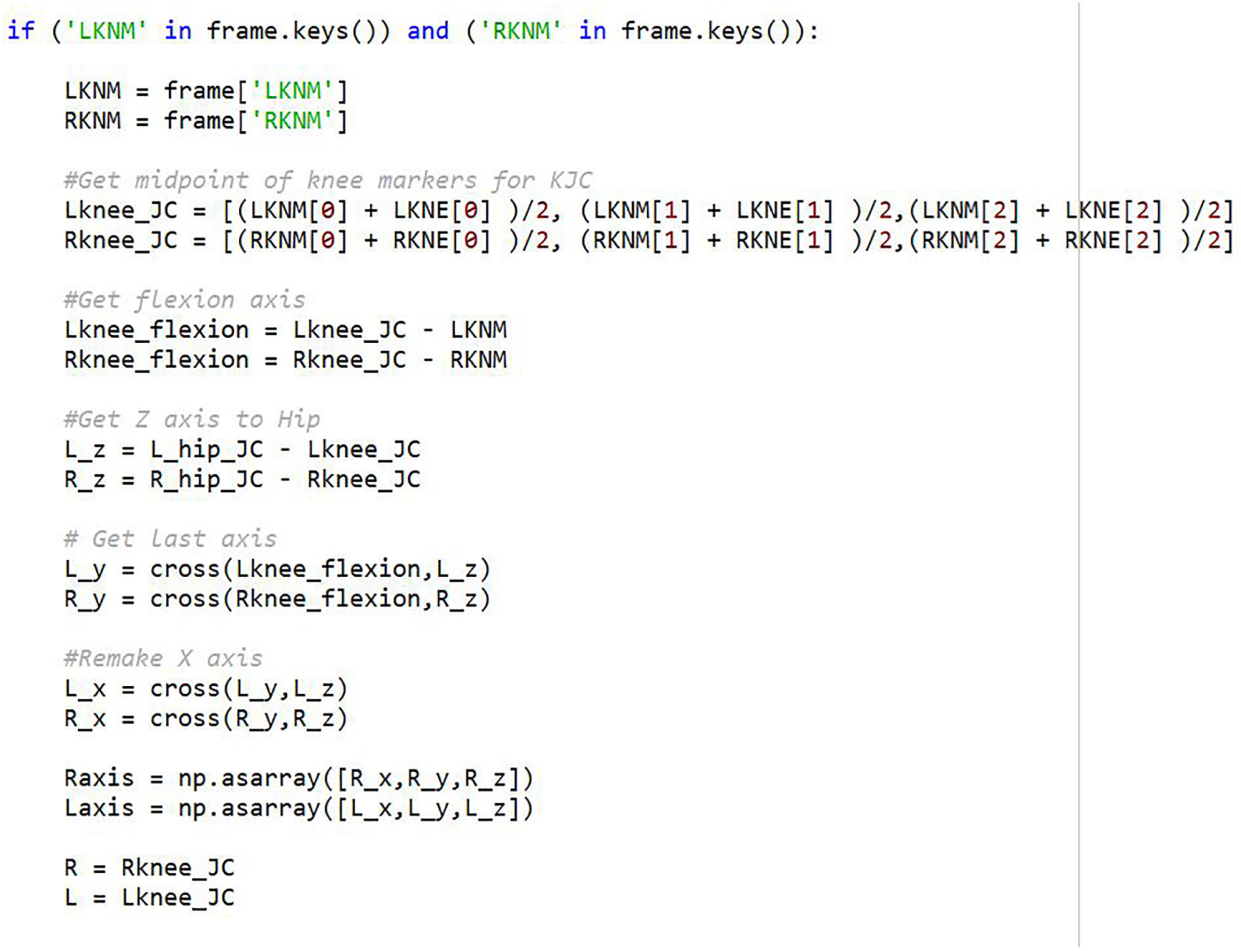
Implementation using knee medial marker. If a Medial marker is detected, the code will use it to determine the knee joint center and axis.

It is also possible to determine the knee joint center locations using the method proposed by Stief [22]. In the code, the presence of a medial knee marker (R/L KNM) determines if the Stief method is implemented. The midpoint between medial and lateral markers is used as the joint center with the vector defined as the midpoint to the medial marker. Fig 12 shows the implementation in the pyCGM system. As the originally described method in [22] did not give exact details, the implementation here is approximate. However, modification of the method is simple.

## 4 Discussion

### 4.1 Summary

This paper presents experimental data for high performance computing of the CGM used for calculating joint kinematics. It demonstrates three methods; specifically, the use of HPC for a single large file of motion capture data by distributing frames to be calculated on separate cores and across nodes, and the distribution of frames across cores on a consumer grade desktop, was investigated. Second, large calculations were performed on a single dataset to derive valuable information about the model, such as the case of subject measurement errors, through the distribution of data across cores.

While many alternatives and modified versions of the CGM exist, this paper focuses on Vicon’s CGM implementation via the plug-in-gait modeler distributed in Vicon Nexus software [10] due to the large number of studies and laboratories that utilize it. As this implementation is based on direct kinematics and uses a single static calibration file, the computation time can easily be reduced through parallelization, an aspect that has been largely left out of discussions on joint kinematics models.

The choice to implement the CGM in Python was made primarily due to the portability between operating systems and the wide acceptance of Python for scripting purposes in the scientific community [38]. The interpreted language and easily understood syntax continues to promote the open source aspect of the work.

As much of the data reported in the literature is focused on the lower body (due to a focus on gait), this paper focused on the lower body. While the upper body has been developed in pyCGM and is included in the computational time, the lack of data relating to marker placement and skin deformation of the upper body makes it difficult to compare the significance of subject measurement errors.

### 4.2 Kinematic validation

There was a strong agreement between pyCGM and Vicon CGM joint kinematic outputs, confirming a correct implementation of the CGM. Small differences observed may be due to numerical implementation differences or rounding differences. Importantly, in the Vicon CGM, the method used to compute the knee and ankle joint center is unclear; however, here, an exact formulation based on the Rodrigues rotation formula [37] is presented. Moreover, the original work of Kadaba [12] specifies the use of the arcsin function which would result in erroneous joint angles during motions greater than 90 degrees. In the pyCGM code, the arctan function is used to solve this problem. It is likely that a similar correction is coded into the Vicon CGM; however, the “black box” nature of the software makes exploration of its underlying code impossible. The flexibility of python and straightforward code of pyCGM allows researchers to easily view, modify, and expand the CGM.

### 4.3 Computation performance

The non-optimized Direct Kinematic (DK) method allows for a frame-by-frame calculation for joint kinematics and, as such, provides an opportunity for easy HPC implementation to assist in the kinematic processing. Similarly, the portability and modularity provided by pyCGM allows researchers to quickly modify the parameters of the model and distribute workloads of either frame-by-frame or trial-to-trial calculations to an HPC. These calculation times are dramatically improved by moving from a classical desktop setup to an HPC, as detailed in the results section.

As a stand-alone compiled program, comparison against Nexus also demonstrates the real speed benefit of parallelization as the interpreted python code is significantly faster than the compiled code of Nexus. At the same time, under utilizing the parallelized methods by reducing the number of cores used has an adverse effect on computation time. While the HPC is orders of magnitude faster than Nexus when using multiple nodes, the use of only 1 core shows significantly slower processing times. This is attributed to the lower clock speed of processors containing large numbers of cores.

However, challenges exist for the type of large databases of motion capture data that can fully take advantage of HPC. The first is the need to improve the ability to track people in an efficient way. While devices such as the Microsoft Kinect may allow for cheaper 3D reconstructions, a more relevant advancement would be the replacement of reflective markers and infrared cameras with a regular RGB camera with tracking markers, such as QR tags, that can be easily implemented on-site in a variety of cases while taking advantage of the research already done on joint landmark kinematic calculations. In the laboratory, more robust gap-filling and auto-labeling technology using the current motion capture systems would also greatly improve the efficiency and bring the use of HPC to a more common audience.

### 4.4 Subject measurement error

The primary, or axis of most importance during an analysis, such as knee flexion during a squat motion, is central to understanding the importance of subject measurements. As seen in the experimental data, during a squat motion the flexion axis in both left and right knee axis were almost never the source of the largest error. As such, while subject measurements have an effect on joint angles, hip and knee flexion are the least affected and may still provide the most valid results. The percentages of error displayed in Table 4 are based on the data found in [23] in which the maximum deviation from 7 laboratories in leg length measurements was 25 mm, knee diameter of 5 mm, and ankle diameter of 10 mm.

While accurate subject measurements are required for the CGM, the importance of these measurements should be considered within the context of other possible sources of angle error. In [39], 10 mm of marker placement error resulted in 6.2 degrees of error in knee rotation and 7.6 degrees in ankle rotation. Comparatively, the 10% change in knee width shown in Table 4 translates to 10.5 mm. This results in a 2.0 degree error in knee rotation and a 3.8 degree error in ankle flexion. While the kinematic model and subject motion from [39] slightly vary from those used in this research, this comparison should give researchers a basis for determining both which aspects of subject preparation are most vital and which aspects of the CGM new models should overcome. In a more general sense, the results suggest that subject measurements can have a significant impact on joint angles. For example, a 50 mm error (5%) in the measurement of leg length, results in over 3 degrees of hip rotation error). In practice, such a large error in leg length may not be likely for experienced users of the CGM (c.f [23] where 25 mm was observed across laboratories), suggesting that more attention should be given to marker placement. Subject measurement errors within the range observed by [23] result in clinically negligible angle errors (less than 2 degrees) [40].

### 4.5 CGM modifications

Past work has shown that rather than completely new models, modifications to existing models provide familiarity and improved accuracy. In [22], the knee joint center estimation was improved by using medial markers. This method was easily integrated into the pyCGM code by adding 18 lines of code directly in the knee joint calculation function with no removal or other modifications necessary as the code switches to this method when medial knee markers are detected. Although this method requires additional markers, the overall marker set remains the same. Without additional markers, the hip joint center estimation can be improved with the Harrington method [41]. This method has been implemented through 9 lines of code in the hip axis calculation, 5 lines of code in the static calibration, and 1 line of code in the execution file which acts as the argument to switch between hip joint center methods. Likewise, [42] shows an improved knee joint center through the adjustment of the thigh offset. Given the open-source nature of pyCGM, interested users could implement this change in the future.

### 4.6 Future directions

The numerous studies for validating the results during gait, the wide usage, and the deep understanding of the model [43] remain important standards for the CGM. In a large field in which multiple methods for calculating joint kinematics are possible, the use of standards for validated models is of great importance.

Since the creation of the original CGM, new methods for computing joint kinematics have been introduced, with the most relevant works modifying the CGM to be used with kinematic fitting and optimizations. C-motion Visual 3D offers a version of the CGM that uses optimization methods through inverse kinematics [44]. Likewise, methods such as the optimized lower-limb gait analysis (OLGA) use inverse kinematics by global optimization [3]. However, these methods often have implications for computational complexity, as in the case of OLGA, in which more than 50 frames of data are required to be considered for good convergence [4], making parallelization a more complex task and the overall computation time much larger. Furthermore, the use of inverse kinematics over direct kinematics is not a guarantee for more accurate results, as research has found that the anatomical model used in the study has a greater effect on kinematics than the different computational methods [5]. Similarly, 6 DOF models such as CAST have also been introduced [6]. However, there is not a strong argument for the use of this model, as similar issues with the CGM exist in CAST [7]. Likewise, the CGM is not without critics and drawbacks, however; as models aim to improve the accuracy of joint angle calculations over the CGM, the effect of subject measurements in errors must be understood.

Beyond the work presented here, there is a need for new computational models to take into account spatial and time complexity, as these are directly related to the impact and implementation possibilities throughout the biomedical industry and other fields. Real-time algorithms for single subject analysis have been developed, which creates implications for clinician-patient interaction [45]. When considering least squares optimization and regression-based methods in the dynamic trial calculation, it is not possible to implement real-time calculations, something that may be useful in a clinical setting for doctor-patient interaction. At the same time, these methods may benefit from HPC when parallelization or distribution is possible. Additionally, the number of markers being recorded has a significant impact on the storage required for these large datasets, hindering data sharing and distribution. However, further research into the actual computational time increase required by optimization methods would help define these limitations.

Applications for computation of large databases of motion capture data extend from biomechanics and robotics to other, less obviously related fields, such as architecture. In architecture, understanding human movement and movement abilities is important for design and necessary in order to move from prescriptive to performative design criteria [46]. In general, any field relying on interactions with humans and movement will at some point need to address the computational efficiency of large scale calculations for analysis.

Finally, further work involving subject measurements in the CGM can shed light on how significant other measurements beyond leg length, knee width, and ankle width are. This may also lead to the ability for subject measurements to be derived directly from marker locations during the static calibration, which would remove the need for storing patient specific data while maintaining a reasonable margin of error.

## Supporting information

S1 Fig Joint center calculation of the knee. For the knee joint center, the calculation is from the thigh marker (a), hip joint center (b), and knee marker (c). The intent is to find the plane in which all markers lay, with half the knee width (kw) being used in the calculation.
(TIF)

S2 Fig Sample code of pyCGM. The python code provides comparison between the mathematics and code that is easy to read and understand. The function receives three marker positions and half of the knee width measurement. The return value is the cartesian location of the calculated joint center. Ease of understanding the code is an important aspect of pyCGM, and as such, the steps are divided clearly so that users can both understand and modify the code to suit their needs.
(TIF)

S3 Fig Flexion beyond 90 degrees using arcsin and arctan. Motion capture data of a sitting motion in which the Knee bends to 90 degrees. While this function was intended for use in gaits that would not commonly have a 90 degree flexion, the widespread use of the CGM includes researchers using it for purposes beyond typical gait.
(TIF)

S1 Equation Equation of the knee joint calculation using Rodrigues rotation formula. (PDF)

S1 Table Results of single dynamic trial scaled on HPC. The Sum column is the sum of the times required for each step in the calculation process. The Total column is the recorded start to finish time, with the difference shown in the last column. This difference includes communication time between cores and nodes, as can be seen from the increased difference when the calculation moved from 1 node to 2 nodes.
(PDF)

S2 Table Computational performance of kinematic calculations for multiple variations of a dynamic trial on the HPC. The average, maximum, and minimum times for each core to complete the calculations are shown. Additionally, the longest time for any core to complete all calculations is shown. Loading data was all done on the initial core. Saving the results, dynamic trial calculation, and static trial calculation times are from every core. The sum of these calculations and the total time recorded from the first node differ mostly due to data transfer between nodes.
(PDF)

S1 File Output from the HPC. (ZIP)

S2 File Breakdown of subject measurement errors. (XLSX)

## References

[1] Baker R. The history of gait analysis before the advent of modern computers. Gait Posture. 2007;26(3):331–242. doi: 10.1016/j.gaitpost.2006.10.014 17306979 10.1016/j.gaitpost.2006.10.014

[2] Lewis JL, Lew WD. A note on the description of articulating joint motion. Journal of Biomechanics. 1977;10(10):675–678. doi: 10.1016/0021-9290(77)90067-7 591522 10.1016/0021-9290(77)90067-7

[3] Roren L, Tate P. A new lower body model using global optimisation techniques. Gait Posture. 2002;16(Suppl 1):S14–5.

[4] Charlton IW, Tate P, Smyth P, Roren L. Repeatability of an optimised lower body model. Gait and Posture. 2004;20(2):213–221. doi: 10.1016/j.gaitpost.2003.09.004 15336293 10.1016/j.gaitpost.2003.09.004

[5] Kainz H, Modenese L, Lloyd D, Maine S, Walsh J, Carty C. Joint kinematic calculation based on clinical direct kinematic versus inverse kinematic gait models (Ms. Ref. No.: BM-D-15-00779 Rev. 1). Journal of Biomechanics. 2016; p. 1–12. 10.1016/j.jbiomech.2016.03.052 27139005

[6] Cappozzo a, Catani F, Della Croce U, Leardini a. Position and orietnation in space of bones during movement. Clin Biomech. 1995;10(4):171–178. 10.1016/0268-0033(95)91394-t 11415549

[7] Collins TD, Ghoussayni SN, Ewins DJ, Kent JA. A six degrees-of-freedom marker set for gait analysis: repeatability and comparison with a modified Helen Hayes set. Gait & posture. 2009;30(2):173–80. doi: 10.1016/j.gaitpost.2009.04.004 19473844 10.1016/j.gaitpost.2009.04.004

[8] Kadaba M, Ramakrishnan H, Wootten M, Gainey J, Gorton G, Cochran G. Repeatability of kinematic, kinetic, and electromyographic data in normal adult gait. Journal of Orthopaedic Research. 1989;7(6):849–860. doi: 10.1002/jor.1100070611 2795325 10.1002/jor.1100070611

[9] Plug-in Gait manual v1; Accessed, Aug 22nd 2014. Available from: http://www.irc-web.co.jp.

[10] Nexus; 2014. Available from: https://www.vicon.com/downloads/core-software/nexus/nexus-185-installer.

[11] Sutherland DH. The evolution of clinical gait analysis. Part II Kinematics. Gait Posture. 2002;16(2):159–79. 12297257 10.1016/s0966-6362(02)00004-8

[12] Kadaba MP, Ramakrishnan HK, Wootten ME. Measurement of lower extremity kinematics during level walking. Journal of orthopaedic research: official publication of the Orthopaedic Research Society. 1990;8(3):383–92. doi: 10.1002/jor.1100080310 2324857 10.1002/jor.1100080310

[13] Grood ES, Suntay WJ. A joint coordinate system for the clinical description of three-dimensional motions: application to the knee. Journal of biomechanical engineering. 1983;105(2):136–44. doi: 10.1115/1.3138397 6865355 10.1115/1.3138397

[14] Groen BE, Geurts M, Nienhuis B, Duysens J. Sensitivity of the OLGA and VCM models to erroneous marker placement: Effects on 3D-gait kinematics. Gait and Posture. 2012;35(3):517–521. doi: 10.1016/j.gaitpost.2011.11.019 22245226 10.1016/j.gaitpost.2011.11.019

[15] Gorton GE, Hebert DA, Gannotti ME. Assessment of the kinematic variability among 12 motion analysis laboratories. Gait and Posture. 2009;29(3):398–402. doi: 10.1016/j.gaitpost.2008.10.060 19056271 10.1016/j.gaitpost.2008.10.060

[16] France L, Nester C. Effect of errors in the identification of anatomical landmarks on the accuracy of Q angle values. Clinical biomechanics (Bristol, Avon). 2001;16(8):710–3. doi: 10.1016/S0268-0033(01)00045-6 10.1016/s0268-0033(01)00045-6 11535354

[17] Lu TW, O’Connor JJ. Bone position estimation from skin marker co-ordinates using global optimisation with joint constraints. Journal of Biomechanics. 1999;32(2):129–134. doi: 10.1016/S0021-9290(98)00158-4 10052917 10.1016/s0021-9290(98)00158-4

[18] Holden JP, Orsini JA, Siegel KL, Kepple TM, Gerber LH, Stanhope SJ. Surface movement errors in shank kinematics and knee kinetics during gait. Gait and Posture. 1997;5(3):217–227. doi: 10.1016/S0966-6362(96)01088-0

[19] Leardini A, Chiari A, Della Croce U, Cappozzo A. Human movement analysis using stereophotogrammetry Part 3. Soft tissue artifact assessment and compensation; 2005. 10.1016/j.gaitpost.2004.05.002 15639400

[20] Davis RB, Ounpuu S, Tyburski D, Gage JR. A gait analysis data collection and reduction technique. Human Movement Science. 1991;10(5):575–587. doi: 10.1016/0167-9457(91)90046-Z

[21] Harrington ZABLSEYZTT M E. Prediction of the hip joint centre in adults, children, and patients with cerebral palsy based on magnetic resonance imaging. Journal of Biomechanics. 2007;40(3):595–602. doi: 10.1016/j.jbiomech.2006.02.003 16584737 10.1016/j.jbiomech.2006.02.003

[22] Stief F, Böhm H, Michel K, Schwirtz A, Döderlein L. Reliability and accuracy in three-dimensional gait analysis: a comparison of two lower body protocols. Journal of applied biomechanics. 2013;29(1):105–111. doi: 10.1123/jab.29.1.105 22813723 10.1123/jab.29.1.105

[23] Benedetti MG, Merlo A, Leardini A. Inter-laboratory consistency of gait analysis measurements. Gait and Posture. 2013;38(4):934–939. doi: 10.1016/j.gaitpost.2013.04.022 23711987 10.1016/j.gaitpost.2013.04.022

[24] Bell G. Supercomputers: The Amazing Race; 2015. MSR-TR-2015-2. Available from: http://research.microsoft.com/apps/pubs/default.aspx?id=238107.

[25] Park S, Park J. Effect of Heel Height and Speed on Gait, and the Relationship Among the Factors and Gait Variables. J Ergon Soc Korea. 2016;35(1):39–52.

[26] Bruijn SM, van Dieën JH, Meijer OG, Beek PJ. Statistical precision and sensitivity of measures of dynamic gait stability. Journal of neuroscience methods. 2009;178(2):327–333. doi: 10.1016/j.jneumeth.2008.12.015 19135478 10.1016/j.jneumeth.2008.12.015

[27] Kurihara K, Hoshino S, Yamane K, Nakamura Y. Optical motion capture system with pan-tilt camera tracking and real time data processing. In: Proceedings 2002 IEEE International Conference on Robotics and Automation (Cat. No.02CH37292). vol. 2. IEEE; 2002. p. 1241–1248. Available from: http://ieeexplore.ieee.org/lpdocs/epic03/wrapper.htm?arnumber=1014713.

[28] Delp SL, Anderson FC, Arnold AS, Loan P, Habib A, John CT, et al OpenSim: Open-source software to create and analyze dynamic simulations of movement. IEEE Transactions on Biomedical Engineering. 2007;54(11):1940–1950. doi: 10.1109/TBME.2007.901024 18018689 10.1109/TBME.2007.901024

[29] Barre A, Armand S. Biomechanical ToolKit: Open-source framework to visualize and process biomechanical data. Computer methods and programs in biomedicine. 2014;114(1):80–7. doi: 10.1016/j.cmpb.2014.01.012 24548899 10.1016/j.cmpb.2014.01.012

[30] Barre A, Turcot K, Bonnefoy-Mazure A, Armand S, Ferrari A, et Al, et al Comparison of biomechanical gait models with the open-source biomechanical toolkit (BTK): Preliminary results. Gait & Posture. 2014;39:S73–S74.

[31] Nakaoka S, Nakazawa A, Kanehiro F, Kaneko K, Morisawa M, Ikeuchi K. Task model of lower body motion for a biped humanoid robot to imitate human dances. In: 2005 IEEE/RSJ International Conference on Intelligent Robots and Systems. IEEE; 2005. p. 3157–3162. Available from: http://ieeexplore.ieee.org/lpdocs/epic03/wrapper.htm?arnumber=1545395.

[32] Pollard NS, Hodgins JK, Riley MJ, Atkeson CG. Adapting human motion for the control of a humanoid robot. In: Proceedings 2002 IEEE International Conference on Robotics and Automation (Cat. No.02CH37292). vol. 2. IEEE; 2002. p. 1390–1397. Available from: http://ieeexplore.ieee.org/lpdocs/epic03/wrapper.htm?arnumber=1014737.

[33] Yamane K, Hodgins J. Simultaneous tracking and balancing of humanoid robots for imitating human motion capture data. In: 2009 IEEE/RSJ International Conference on Intelligent Robots and Systems. IEEE; 2009. p. 2510–2517. Available from: http://ieeexplore.ieee.org/lpdocs/epic03/wrapper.htm?arnumber=5354750.

[34] Wojtusch J, von Stryk O. HuMoD—A versatile and open database for the investigation, modeling and simulation of human motion dynamics on actuation level. In: 2015 IEEE-RAS 15th International Conference on Humanoid Robots (Humanoids). IEEE; 2015. p. 74–79. Available from: http://ieeexplore.ieee.org/lpdocs/epic03/wrapper.htm?arnumber=7363534.

[35] Jackson KR, Ramakrishnan L, Muriki K, Canon S, Cholia S, Shalf J, et al. Performance Analysis of High Performance Computing Applications on the Amazon Web Services Cloud. In: 2010 IEEE Second International Conference on Cloud Computing Technology and Science. IEEE; 2010. p. 159–168. Available from: http://ieeexplore.ieee.org/lpdocs/epic03/wrapper.htm?arnumber=5708447.

[36] Schwartz M. pyCGM; 2016. Available from: https://github.com/cadop/pyCGM.

[37] Cheng H, Gupta KC. An historical note on finite rotations. Journal of Applied Mechanics. 1989;56(1):139–145. doi: 10.1115/1.3176034

[38] Langtangen HP. Python scripting for computational science. vol. 3 Springer; 2006.

[39] Osis ST, Hettinga BA, Macdonald S, Ferber R, GG 3rd, Hebert D, et al Effects of Simulated Marker Placement Deviations on Running Kinematics and Evaluation of a Morphometric-Based Placement Feedback Method. PLOS ONE. 2016;11(1):e0147111 doi: 10.1371/journal.pone.0147111 26765846 10.1371/journal.pone.0147111 PMC4713202

[40] McGinley JL, Baker R, Wolfe R, Morris ME. The reliability of three-dimensional kinematic gait measurements: A systematic review. Gait and Posture. 2009;29(3):360–369. doi: 10.1016/j.gaitpost.2008.09.003 19013070 10.1016/j.gaitpost.2008.09.003

[41] Parker K, Stebbins J, Bates J. Comparing the Harrington and Davis method of hip joint centre localisation for unimpaired and pathological subjects. Gait & Posture. 2014;39:S114–S115. doi: 10.1016/j.gaitpost.2014.04.158

[42] Baker R, Finney L, Orr J. A new approach to determine the hip rotation profile from clinical gait analysis data. Human Movement Science. 1999;18(5):655–667. doi: 10.1016/S0167-9457(99)00027-5

[43] Baker R. Measuring walking: a handbook of clinical gait analysis. Hart Hilary M, editor. Mac Keith Press; 2013.

[44] C-Motion. Visual 3D;. Available from: https://www.c-motion.com/v3dwiki/index.php?title=T.

[45] Van den Bogert AJ, Geijtenbeek T, Even-Zohar O, Steenbrink F, Hardin EC. A real-time system for biomechanical analysis of human movement and muscle function. Medical & biological engineering & computing. 2013;51(10):1069–1077. doi: 10.1007/s11517-013-1076-z 23884905 10.1007/s11517-013-1076-z PMC3751375

[46] Schwartz M. Collaborative and Human Based Performance Analysis In: eCAADe: Models of Computation—Human Factors. vol. 2 Delft: Faculty of Architecture, Delft University of Technology; 2013 p. 365–374.

