# Supplementary figures and images for: The effect of subject measurement error on joint kinematics in the conventional gait model: Insights from the open-source pyCGM tool using high performance computing methods
Source: PLoS One. 2018 Jan 2;13(1):e0189984. doi: 10.1371/journal.pone.0189984 (PMC5749724; doi:10.1371/journal.pone.0189984)

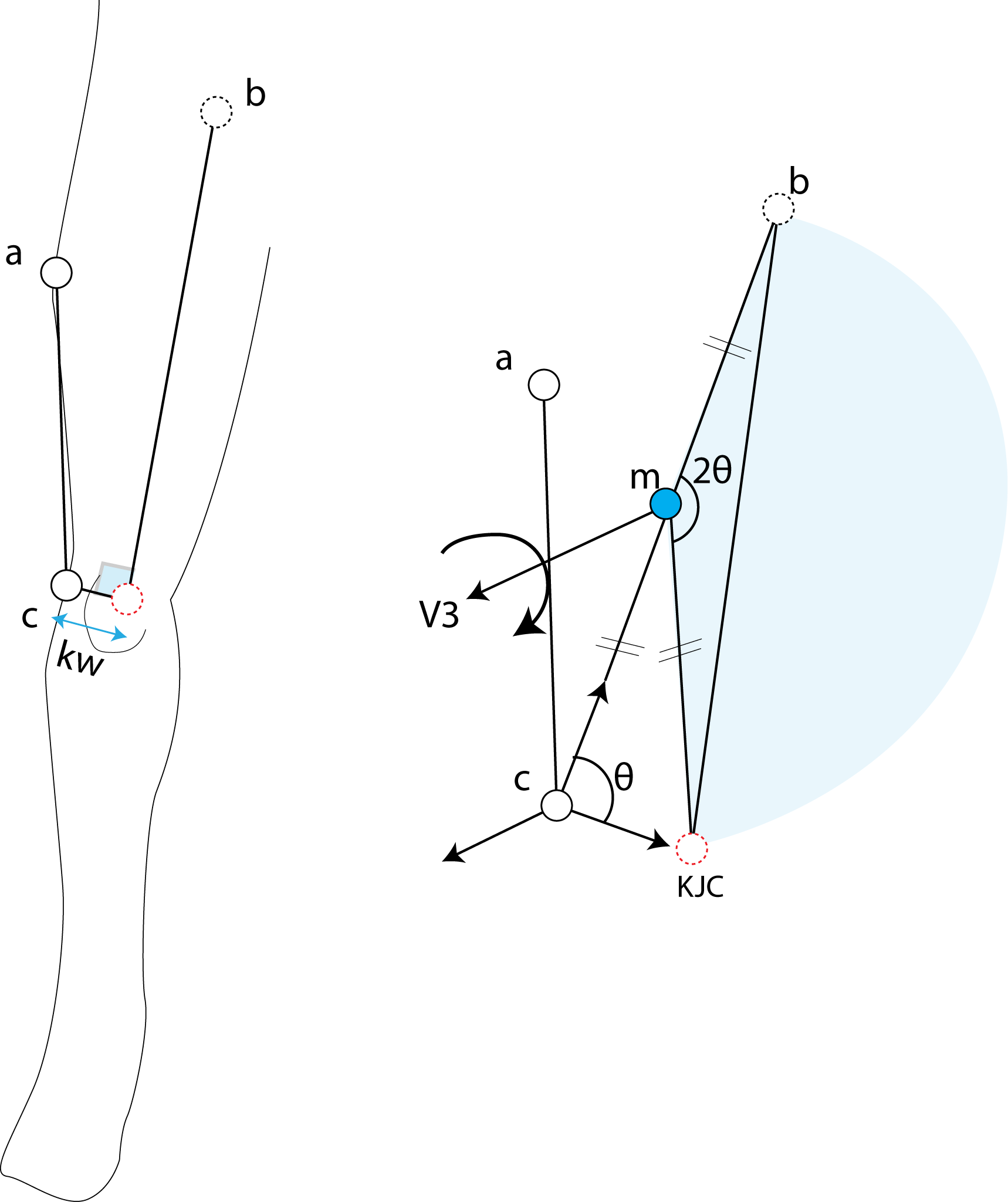

Supplement: S1 Fig — For the knee joint center, the calculation is from the thigh marker (a), hip joint center (b), and knee marker (c). The intent is to find the plane in which all markers lay, with half the knee width (kw) being used in the calculation. (TIF) [file pone.0189984.s001.tif]

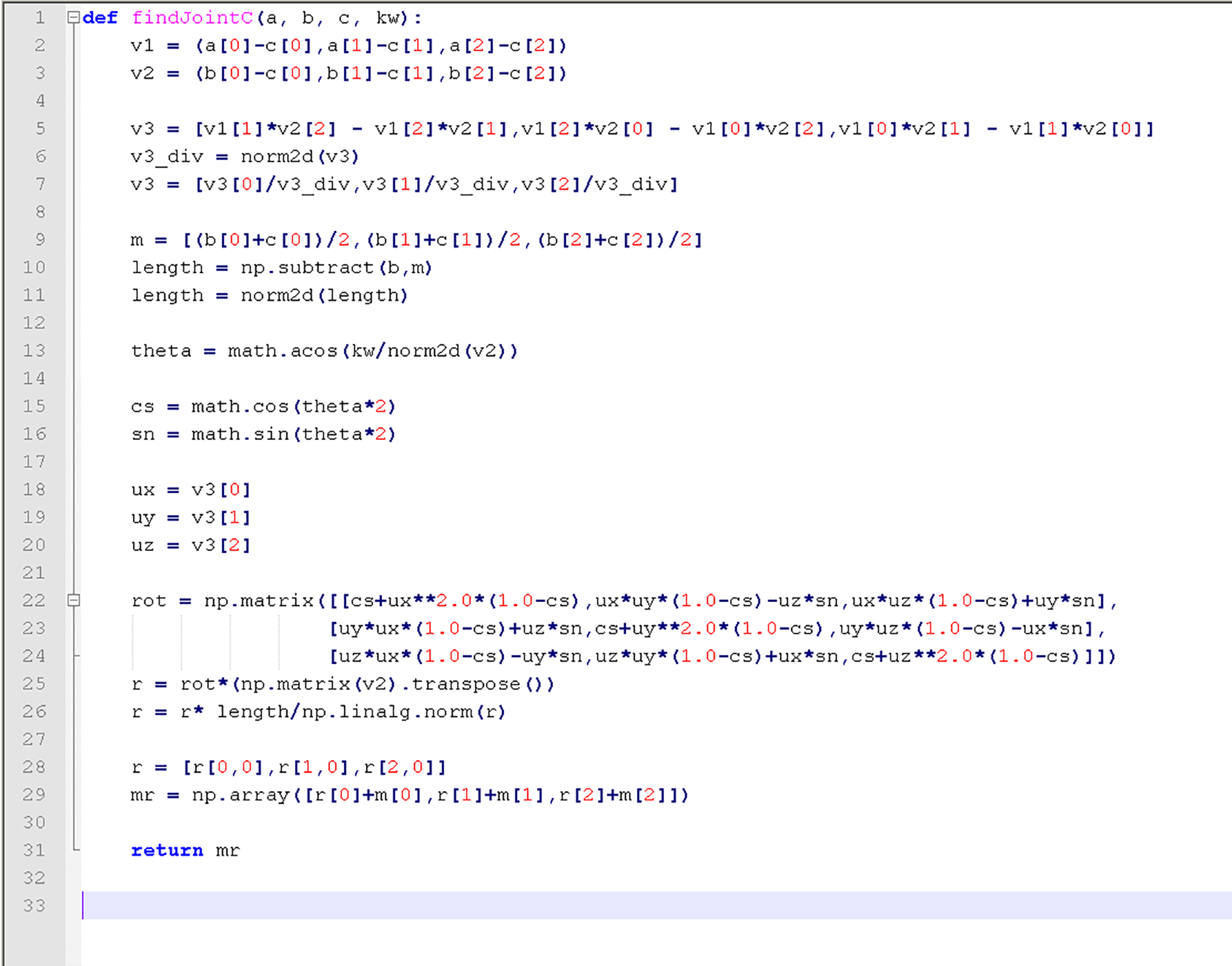

Supplement: S2 Fig — The python code provides comparison between the mathematics and code that is easy to read and understand. The function receives three marker positions and half of the knee width measurement. The return value is the cartesian location of the calculated joint center. Ease of understanding the code is an important aspect of pyCGM, and as such, the steps are divided clearly so that users can both understand and modify the code to suit their needs. (TIF) [file pone.0189984.s002.tif]

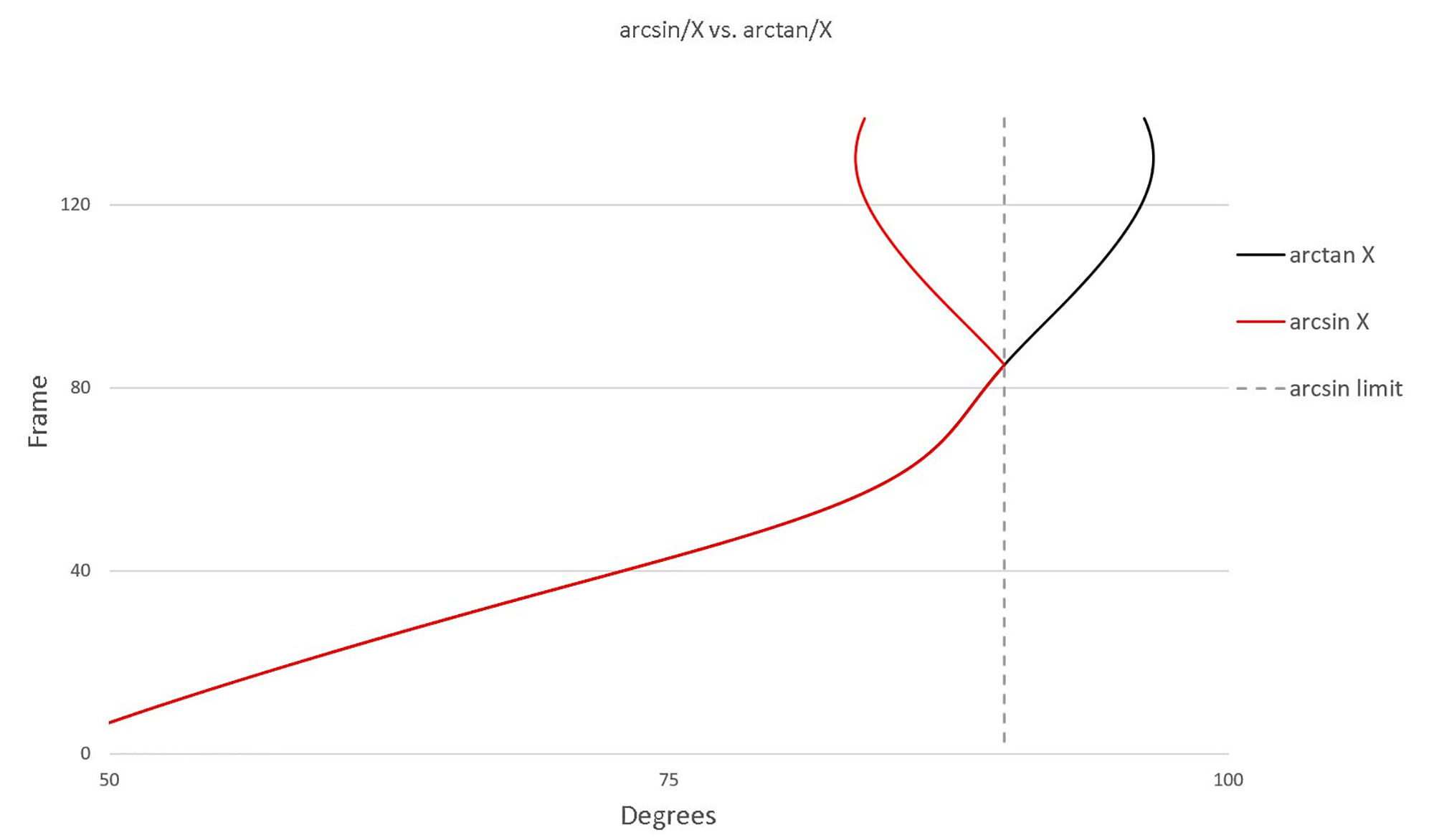

Supplement: S3 Fig — Motion capture data of a sitting motion in which the Knee bends to 90 degrees. While this function was intended for use in gaits that would not commonly have a 90 degree flexion, the widespread use of the CGM includes researchers using it for purposes beyond typical gait. (TIF) [file pone.0189984.s003.tif]
