# Supplementary material for: The effect of subject measurement error on joint kinematics in the conventional gait model: Insights from the open-source pyCGM tool using high performance computing methods
Source: PLoS One. 2018 Jan 2;13(1):e0189984. doi: 10.1371/journal.pone.0189984 (PMC5749724; doi:10.1371/journal.pone.0189984)
Supplement: S1 Equation — (PDF) [file pone.0189984.s004.pdf]

$$\hat{V_1} = \frac{a - c}{\|a - c\|}$$
$$V_2 = b - c$$
$$\hat{V_2} = \frac{V_2}{\|V_2\|}$$
$$\hat{V_3} = \frac{\hat{V_1} \times \hat{V_2}}{\|\hat{V_1} \times \hat{V_2}\|}$$
$$m = \frac{b + c}{2}$$
$$length = \|b - m\|$$

The rotation amount is directly related to the knee width such that,

$$\theta = \cos^{-1} \frac{kw}{\|V_2\|}$$
$$\hat{V_3} = (u_x, u_y, u_z)$$

With the Rodrigues rotation formula using θ to calculate r,

$$R = \begin{bmatrix} u_x^2(1 - cos2\theta) + cos2\theta & u_x u_y(1 - cos2\theta) + u_z sin2\theta & u_x u_z(1 - cos2\theta) - u_y sin2\theta \\ u_y u_x(1 - cos2\theta) - u_z sin2\theta & u_y^2(1 - cos2\theta) + cos2\theta & u_y u_z(1 - cos2\theta) + u_x sin2\theta \\ u_z u_x(1 - cos2\theta) + u_y sin2\theta & u_z u_y(1 - cos2\theta) - u_x sin2\theta & u_z^2(1 - cos2\theta) + cos2\theta \end{bmatrix}$$

$$r = R \times \hat{V_2}$$
$$\hat{r} = \frac{r}{\|r\|}$$
$$mr = \hat{r} \times length + m$$
