# Supplementary material for: The effect of subject measurement error on joint kinematics in the conventional gait model: Insights from the open-source pyCGM tool using high performance computing methods
Source: PLoS One. 2018 Jan 2;13(1):e0189984. doi: 10.1371/journal.pone.0189984 (PMC5749724; doi:10.1371/journal.pone.0189984)
Supplement: S1 Table — The Sum column is the sum of the times required for each step in the calculation process. The Total column is the recorded start to finish time, with the difference shown in the last column. This difference includes communication time between cores and nodes, as can be seen from the increased difference when the calculation moved from 1 node to 2 nodes. (PDF) [file pone.0189984.s005.pdf]

**Nodes**	**Cores**	**Total**		**Load VSK**	**Load Static**	**Load File**	**Calculate Static**	**Calculate Dynamic**	**Save Output**	**Sum**	**Total**	**Diff.**
1	2	2		0.0503	0.0716	3.0562	0.5700	188.3760	7.4869	199.6110	200.3160	0.7050
1	4	4		0.0886	0.0730	3.2968	0.4975	66.8116	7.8153	78.5827	79.3363	0.7536
1	16	16		0.0472	0.0822	3.4435	0.5102	14.7648	8.1782	27.0262	27.7922	0.7660
2	16	32		0.1792	0.0738	3.4434	0.5206	7.8854	8.3757	20.4781	21.3224	0.8443
4	16	64		0.1519	0.1437	3.5840	0.5235	4.4654	8.4629	17.3314	18.1792	0.8479
8	16	128		0.0495	0.0932	3.5046	0.5191	3.0400	8.3984	15.6049	16.4495	0.8445
16	16	256		0.0516	0.0771	3.5749	0.5209	2.2210	8.4207	14.8662	15.7137	0.8475
