# Supplementary material for: The effect of subject measurement error on joint kinematics in the conventional gait model: Insights from the open-source pyCGM tool using high performance computing methods
Source: PLoS One. 2018 Jan 2;13(1):e0189984. doi: 10.1371/journal.pone.0189984 (PMC5749724; doi:10.1371/journal.pone.0189984)
Supplement: S2 Table — The average, maximum, and minimum times for each core to complete the calculations are shown. Additionally, the longest time for any core to complete all calculations is shown. Loading data was all done on the initial core. Saving the results, dynamic trial calculation, and static trial calculation times are from every core. The sum of these calculations and the total time recorded from the first node differ mostly due to data transfer between nodes. (PDF) [file pone.0189984.s006.pdf]

Total Cores		Load VSK	Load Static	Load File	Save Output	Calculate Dynamic	Calculate Static	Sum	Total Time
800	**Average**	0.0868	0.0782	0.6659	2.7680	179.6739	5.3117	188.5845	203.9393
1600	**Average**	0.0729	0.0638	0.8421	1.6890	89.7404	3.4361	95.8445	104.2829
800	**Max**				3.7644	193.0373	5.6910	202.4926	
1600	**Max**				2.4327	96.9163	3.7222	103.0711	
800	**Min**				2.6003	172.7741	5.0137	180.3882	
1600	**Min**				1.2044	79.8708	2.5320	83.6073	
800	**Longest core**				2.8633	187.5211	5.4832	195.8676	
1600	**Longest core**				1.8822	94.1235	3.4251	99.4309	
